# Efficacy and Safety of Neoadjuvant Immunotherapy plus Chemotherapy versus Neoadjuvant Chemotherapy in Non‐Small Cell Lung Cancer Treatment: A Mixed Method Meta‐Analysis Based on Global Randomized Controlled Trials

**DOI:** 10.1002/mco2.70211

**Published:** 2025-05-31

**Authors:** Shen Li, Chenhao Xu, Yuanling Meng, Yiyang Li, Jiaqing Yang, Junxuan Du, Fan Zhang, Hu Liao, Xuelei Ma

**Affiliations:** ^1^ Department of Biotherapy West China Hospital and State Key Laboratory of Biotherapy Sichuan University Chengdu China; ^2^ West China School of Medicine West China Hospital Sichuan University Chengdu Sichuan China; ^3^ West China School of Stomatology Sichuan University Chengdu Sichuan China; ^4^ Shihezi University School of Medicine Shihezi University Xinjiang China; ^5^ Health Management Center General Practice Medical Center West China Hospital Sichuan University Chengdu Sichuan China; ^6^ Department of Thoracic Surgery West China Hospital Sichuan University Chengdu China

**Keywords:** efficacy, immunotherapy, neoadjuvant therapy, non‐small cell lung cancer, safety

## Abstract

This study explores the efficacy and safety of combining immunotherapy with chemotherapy for neoadjuvant non‐small cell lung cancer (NSCLC) treatment. Despite the rapid advancement of immunotherapy, direct evidence comparing it with chemotherapy alone is limited. Following PRISMA guidelines, we analyzed global phase II and III randomized controlled trials data. Our meta‐analysis included nine trials with 3431 participants, showing that combined treatment significantly improved event‐free survival, pathological complete response, major pathological response, surgical acceptance, and R0 resection rates in NSCLC compared with chemotherapy alone. Safety analysis revealed similar all adverse event incidences between treatments, while Grade ≥3 adverse events were higher than those in neoadjuvant chemotherapy. Network meta‐analysis confirmed the benefits of different immunotherapeutic drugs and efficacy in different subgroups. The study's high‐quality evidence suggests that neoadjuvant immunotherapy plus chemotherapy should be considered in clinical practice for NSCLC, with further research needed to optimize treatment strategies and improve patient outcomes.

## Graphical abstract

This study evaluates the efficacy and safety of neoadjuvant immunotherapy combined with chemotherapy for NSCLC. Data from nine trials (3431 participants) reveal significant improvements in event‐free survival, pathological response, and surgical outcomes compared with chemotherapy alone, with slight treatment‐related adverse events increased. These findings support integrating combination therapy into clinical practice.

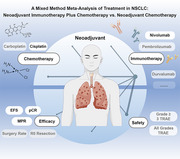

## 1 Introduction

Lung cancer remains one of the leading causes of cancer‐related mortality worldwide, with non‐small cell lung cancer (NSCLC) accounting for 80–85% of all cases. As most NSCLC patients are diagnosed at an advanced stage, the 5‐year survival rate remains low, presenting a significant clinical challenge in the effective treatment of late‐stage NSCLC [1]. Neoadjuvant therapy, administered before primary surgery, aims to reduce tumor size to facilitate easier surgical resection or to render previously unresectable tumors operable. This approach not only enhances the likelihood of successful surgery but also provides guidance for subsequent treatments and reduces the risk of metastasis [2]. Currently, chemotherapy is the primary neoadjuvant treatment for NSCLC and has shown efficacy in prolonging patient survival. However, its effectiveness is limited and is often accompanied by significant side effects [3]. Consequently, immunotherapy, an emerging treatment modality that has demonstrated safety and efficacy across various cancers, has recently been introduced as a component of neoadjuvant treatment for NSCLC. The combination of immunotherapy with chemotherapy is expected to leverage the cytotoxic effects of chemotherapy along with the immune system's antitumor capabilities, further reducing tumor size, improving surgical outcomes, and enhancing long‐term prognosis. This combined neoadjuvant strategy has thus become a key focus of recent research, aiming to extend patient survival and reduce recurrence rates [4].

The Checkmate‐159 study, the large‐scale phase II LCMC3 study, and the Chinese ChiCTR‐OIC‐17013726 study have essentially confirmed the safety and efficacy of neoadjuvant immunotherapy in NSCLC patients, achieving a significant major pathological response (MPR) rate of 19–45% [5, 6]. It was demonstrated that there was a superior efficacy of combined immunotherapy and chemotherapy over chemotherapy alone in lung cancer, as evidenced by the KEYNOTE‐189, KEYNOTE‐407, and IMpower130 double‐blind phase III clinical trials. Subsequently, phase II studies such as the NADIM trial and NCT02716038 have also conducted single‐arm clinical trials on neoadjuvant immunotherapy combined with chemotherapy in lung cancer patients, verifying their safety and efficacy [7, 8, 9, 10, 11].

Since the initiation of dual‐arm clinical trials of neoadjuvant immunotherapy plus chemotherapy versus chemotherapy alone in lung cancer, reports on the efficacy and safety of neoadjuvant chemotherapy plus immunotherapy have been emerging [12]. However, there is still a lack of higher‐level evidence to prove whether neoadjuvant immunotherapy plus chemotherapy offers superior safety and efficacy compared with neoadjuvant chemotherapy alone, especially in MPR and surgery‐related outcomes. Therefore, this study aims to systematically evaluate the differences in efficacy and safety between neoadjuvant immunotherapy combined with chemotherapy and chemotherapy alone in patients with NSCLC, as well as to further compare the relative advantages of different immunotherapeutic agents through a network meta‐analysis. Drawing upon higher‐level evidence, our main findings confirm the synergistic benefits and acceptable safety profile of neoadjuvant immunotherapy with chemotherapy and reveal the potential advantages of dual immunotherapy regimens.

## 2 Result

### 2.1 The Characteristics of Included Study

**FIGURE 1:**
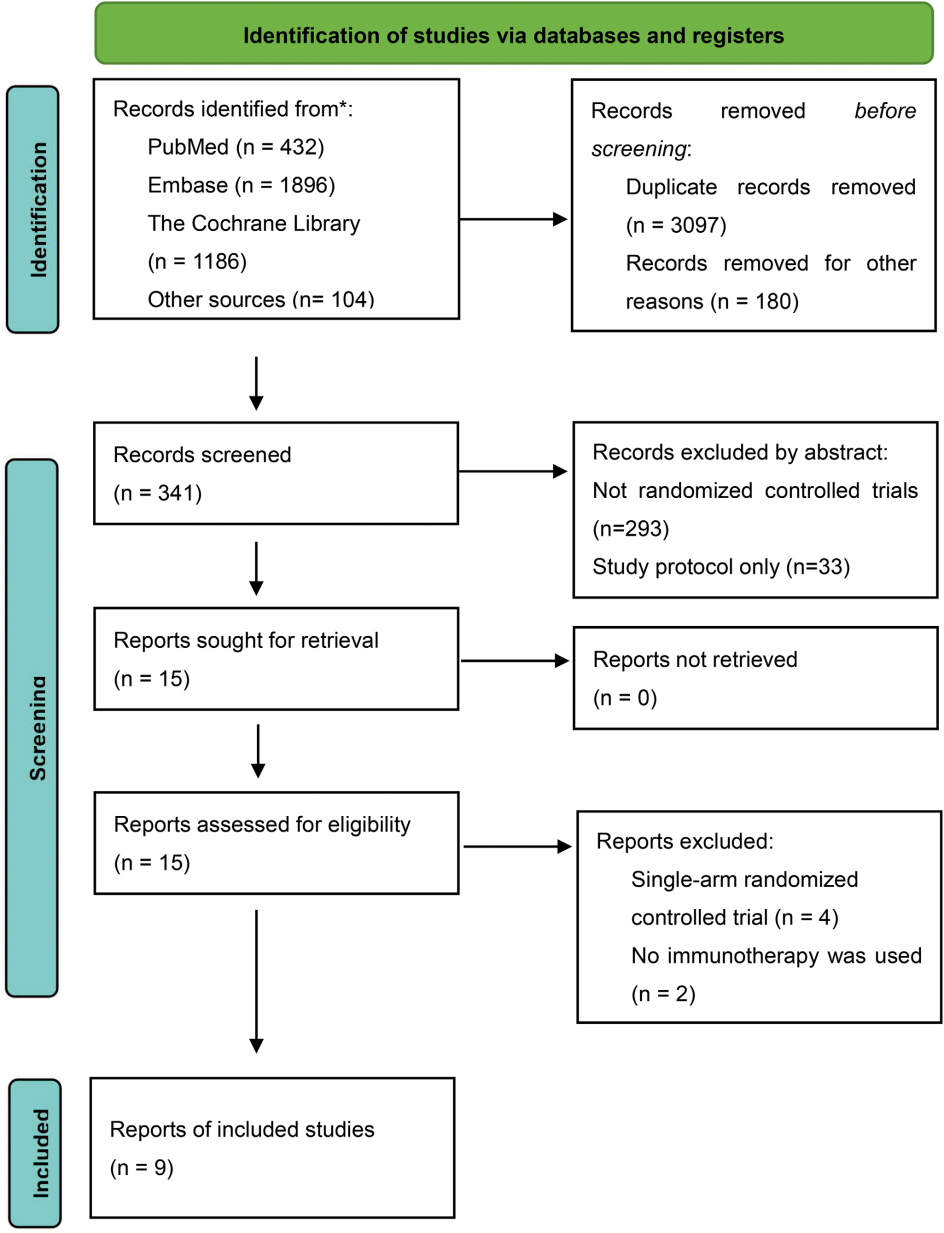
Study selection.

Nine trials met the inclusion criteria, encompassing a total of 3431 participants [12, 13, 14, 15, 16, 17, 18, 19]. The median age was over 60 years, with more than 50% being male and over 85% of the patients being smokers. The NSCLC diagnoses were primarily stage III or higher. The basic characteristics of the included studies are presented in Tables 1 and 2. Eight studies were included in the pooled meta‐analysis, as the study on ipilimumab and nivolumab plus chemotherapy was a comparison with nivolumab plus chemotherapy, rather than just neoadjuvant chemotherapy, and was thus only included in the network meta‐analysis (Figure 1).

**TABLE 1:** The demographic and clinical characteristics of included studies.

Study, year	Country	Clinical trial	Media*n* age (year)	T stage, 1/2/3/4 (*N*)	Stage, (≤II)/(≥III)	Squamous histology (%)	Male/female (*N*)	Smoking/never (*N*)	ECOG score, 0/1	PD‐L1 expression level, (<1%)/(1–49%)/(≥50%)
Patrick, 2022	America	NCT02998528	64	NA	65/113	48.60%	128/51	160/19	124/55	78/51/38
			65	NA	62/115	53.10%	127/52	158/20	117/62	77/47/42
Heather, 2023	America	NCT03425643	63	55/106/121/115	118/279	43.10%	279/118	343/54	253/144	138/127/132
			64	61/126/109/104	121/279	43.30%	284/116	353/47	246/154	151/115/134
Tina, 2023	America	NCT03158129	69.5	NA	11/11	18.20%	10/12	17/5	10/12	NA
			63.1	NA	9/13	18.20%	15/7	17/5	16/6	NA
Mariano, 2023	Spain	NCT03838159	65	12/16/15/14	NA	NA	36/21	52/5	31/26	NA
			63	4/7/6/12	NA	NA	16/13	29/0	16/13	NA
John, 2023	America	NCT03800134	65	44/97/128/97	104/261	46.20%	252/114	315/51	251/115	122/135/109
			65	43/108/129/94	110/263	51.10%	278/96	316/56	255/119	125/142/107
Jie Lei, 2023	China	NCT04338620	61	2/19/16/6	0/43	62.80%	34/9	31/12	41/2	7/NA/NA
			61	4/18/13/10	0/45	71.10%	40/5	37/8	43/2	8/NA/NA
Shun Lu, 2023	China	NCT04158440	62	NA	0/202	77.80%	181/21	172/28	NA	51/69/64
			61	NA	0/202	77.80%	189/13	181/21	NA	54/68/64
Tina, 2024	America	NCT04025879	66	NA	81/146	50.66%	167/62	212/17	147/82	93/83/45
			66	NA	81/149	50.86%	160/72	205/27	141/91	93/76/52
Dongsheng Yue, 2023	China	NCT04379635	62	NA	93/132	79.20%	205/21	192/34	142/83	NA
			63	NA	93/132	77.10%	205/22	188/39	154/73	NA

Abbreviation: CI, confidence interval; CT, chemotherapy; ECOG, eastern cooperative oncology group; EFS, event‐free survival; HR, hazard ratio; MPR, major pathological response; *N*, number; NA, not available; NR, not reached; NSCLC, non‐small‐cell lung cancer; OS, overall survival; PCR, pathological complete response; TRAEs, treatment‐related adverse events.

**TABLE 2:** The clinical outcomes of included studies.

Study, year	Country	Clinical trial	Treatment	Dose of neoadjuvant/(interval^a^ cycles)	Number	Median EFS, month	HR[CI] for median EFS^a^	Median OS, month	HR[CI] for median OS^a^	Surgery (%)	R0 resection	PCR (%)	MPR (%)	Grade≥3 TRAEs (%)	All grade TRAEs (%)
Patrick, 2022	America	NCT02998528	Nivolumab + CT	360 mg/(3w^a^3)	179	31.6	0.63 [0.43, 0.91]	NR	0.57 [0.30, 1.07, 99.67%CI]	83.24%	83.22%	21.23%	36.9%	33.52%	82.39%
			CT		179	20.8		NR		75.42%	77.78%	2.23%	8.9%	36.93%	88.64%
Heather, 2023	America	NCT03425643	Pembrolizumab + CT	200 mg/(3w^a^4)	397	NR	0.58 [0.46, 0.72]	NR	0.73 [0.54, 0.99]	82.07%	92.00%	18.18%	30.30%	44.95%	96.72%
			CT		400	17		45.5		79.45%	84.23%	4.01%	11.03%	37.34%%	94.99%
Tina, 2023	America	NCT03158129	Nivolumab + CT	360 mg/(3w^a^3)	22	NR	1.28 [0.47, 3.46]	NR	NA	100.00%	90.91%	18.18%	31.82%	45.45%	90.91%
			Ipilimumab and nivolumab + CT	Ipi: 1 mg/kg, just 1 day	22	NR		NR		90.91%	95.00%	18.18%	50.00%	20.00%	90.91%
Mariano 2023	Spain	NCT03838159	Nivolumab +CT	360 mg/(3w^a^3)	57	NR	0.47 [0.25, 0.88]	NR	0.43 [0.19, 0.98]	92.98%	94.34%	36.84%	53.00%	NA	NA
			CT		29	15.4		NR		68.97%	85.00%	6.90%	14.00%	NA	NA
John, 2023	America	NCT03800134	Durvalumab + CT	1500 mg/(3w^a^4)	366	NR	0.68 [0.53, 0.88]	NA	NA	80.60%	94.72%	17.21%	NA	32.51%	86.89%
			CT		374	25.9		NA		80.75%	91.29%	4.28%	NA	32.89%	80.75%
Jie Lei, 2023	China	NCT04338620	Camrelizumab + CT	200 mg/(3w^a^3)	43	NR	0.52 [0.21, 1.29]	NA	NA	93.02%	92.50%	32.56%	65.12%	25.58%	95.35%
			CT		45	NR		NA		93.33%	85.71%	8.89%	15.56%	11.11%	88.89%
Shun Lu, 2023	China	NCT04158440	Toripalimab + CT	240 mg/(3w^a^3)	202	NR	0.40 [0.28, 0.57]	NA	NA	82.18%	95.78%	24.75%	48.51%	63.37%	99.50%
			CT		202	15.1		NA		73.27%	92.57%	9.90%	8.42%	53.96%	98.51%
Tina, 2024	America	NCT04025879	Nivolumab + CT	360 mg/(3w^a^4)	229	NR	0.58 [0.42, 0.81, 97.36%CI]	NA	NA	77.73%	89.3%	25.33%	35.37%	32.46%	89.04%
			CT		232	18.4		NA		76.72%	90.4%	4.74%	12.07%	25.22%	86.96%
Dongsheng Yue, 2023	China	NCT04379635	Tislelizumab + CT	NA	226	NA	NA	NA	NA	NA	NA	40.70%	56.20%	69.47%	99.12%
			CT		227	NA		NA		NA	NA	5.70%	15.00%	65.49%	99.56%

Abbreviations: CI confidence interval; CT, chemotherapy; ECOG, eastern cooperative oncology group; EFS, event‐free survival; NA, not available; HR, hazard ratio; MPR, major pathological response; N, number; NR, not reached; NSCLC, non‐small‐cell lung cancer; OS, overall survival; PCR, pathological complete response; TRAEs, treatment‐related adverse events.

^a^ Defaults to 95% CI unless otherwise noted.

### 2.2 Efficacy of Neoadjuvant Immunotherapy plus Chemotherapy

**FIGURE 2:**
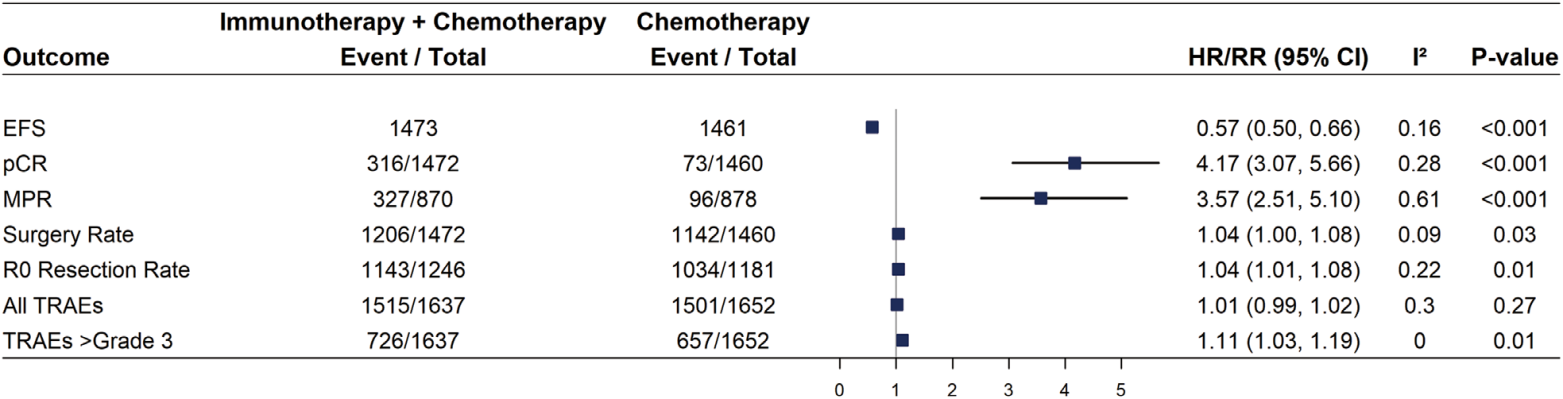
Efficacy and safety of neoadjuvant immunotherapy plus chemotherapy compared with neoadjuvant chemotherapy in pooled meta‐analysis.

The results of the pooled meta‐analysis of event‐free survival (EFS) in patients receiving neoadjuvant immunotherapy plus chemotherapy, compared with neoadjuvant chemotherapy, showed that hazard ratios (HRs) of the median EFS was 0.57 [0.50, 0.66] (*I* ^2^ = 16%, *p* < 0.001) (high‐grade evidence). This suggested that patients receiving the combined therapy had a 43% lower risk of progression or death compared with those receiving chemotherapy alone, demonstrating a substantial improvement in EFS. Furthermore, the relative risks (RRs) for pathological complete response (pCR) and MPR were 4.17 [3.07, 5.66] and 3.57 [2.51, 5.10], respectively, both showing *p* values less than 0.001 (high‐ and moderate‐grade evidence). The results indicated that patients receiving the combined therapy were 4.17 times more likely to achieve a complete pathological response and 3.57 times more likely to achieve a MPR compared with those receiving chemotherapy alone, suggesting a significant improvement in pathological outcomes. The combined neoadjuvant immunotherapy and chemotherapy also slightly outperformed the control group in terms of surgery acceptance rate and R0 resection rate, with RRs of 1.04 [1.00, 1.08] (*I* ^2^ = 9%, *p* = 0.03) (high‐grade evidence) and 1.04 [1.01, 1.08] (*I* ^2^ = 22%, *p* = 0.006) (high‐grade evidence), respectively, suggesting that the combined treatment may lead to better surgical outcomes compared with chemotherapy alone (Figure 2).

### 2.3 Safety of Neoadjuvant Immunotherapy plus Chemotherapy

A meta‐analysis of the incidence rate of all treatment‐related adverse events (TRAEs) for neoadjuvant immunotherapy plus chemotherapy therapy showed a very similar to that of neoadjuvant chemotherapy, with an RR of 1.00 [0.99, 1.02], with *I* ^2^ = 30% and *p* = 0.27 (moderate‐grade evidence). The RR for severe TRAEs (Grade ≥ 3) was 1.11 [1.03, 1.19], and *I* ^2^ = 0%, *p* = 0.007. Although this result showed a slight but not statistically different increase in the incidence of all TRAEs for the neoadjuvant immunotherapy combination regimen, there was a 11% increase in the incidence of severe TRAEs (Figure 2).

### 2.4 Efficacy of Neoadjuvant Immunotherapy Drugs in Network Meta‐Analysis

**FIGURE 3:**
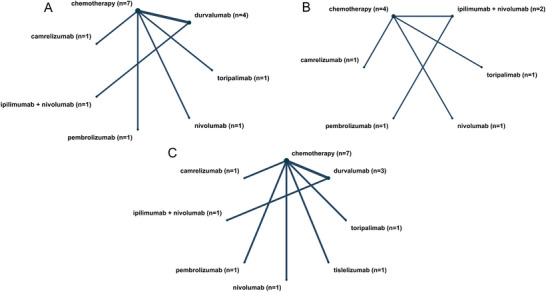
Network diagram in network meta‐analysis. (A) Network diagram of R0 resection, pCR, EFS, and surgery rate; (B) network diagram of MPR; (C) network diagram of all TRAEs and TRAEs ≥ Grade 3.

**FIGURE 4:**
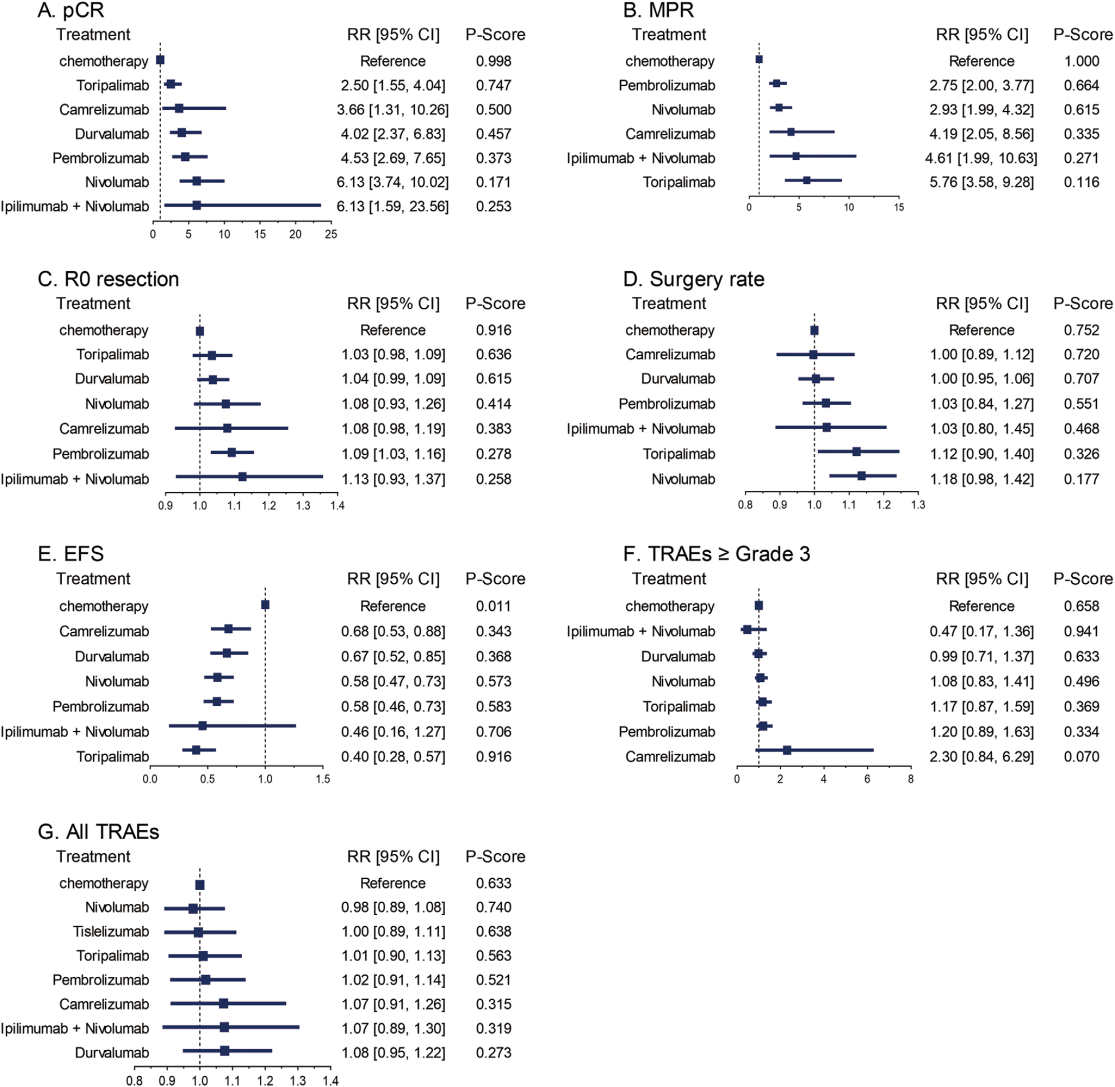
Efficacy and safety of neoadjuvant immunotherapy plus chemotherapy compared with neoadjuvant chemotherapy in network meta‐analysis. (A) The result of pCR; (B) the result of MPR; (C) the result of R0 resection rate; (D) the result of surgery rate; (E) the result of EFS; (F) the result of TRAEs ≥ Grade 3; (G) the result of all TRAEs.

Figure 3 shows a network diagram illustrating the connections between the efficacy and safety outcomes of neoadjuvant immunotherapy and chemotherapy across nine included trials. The network meta‐analysis indicated that, compared with neoadjuvant chemotherapy alone, different neoadjuvant immunotherapy agents demonstrated similar or superior efficacy, including improvements in EFS, pCR, MPR, surgery rate, and R0 resection rate (Figure 4). Notably, the results for EFS, pCR, and MPR further validated the advantage and necessity of adding neoadjuvant immunotherapy agents.

In terms of EFS, almost all combination regimens demonstrated a significantly reduced risk of recurrence or disease progression compared with chemotherapy alone, leading to a significant extension of EFS. Among them, the combination of ipilimumab and nivolumab showed notable clinical significance (RR 0.46 [0.16, 1.27]), though it did not reach statistical significance, potentially due to the sparse network connections. All immunotherapy agents showed some improvement in pCR, with immunotherapy combination therapies demonstrating at least a twofold increase in complete pathological response rates compared with chemotherapy. Similarly, significant improvements were observed in MPR, suggesting substantial potential in reducing tumor burden. Regarding surgery acceptance rate and R0 resection rate, immunotherapy agents did not show a clear advantage but still demonstrated clinical significance, considering that the baseline surgery and R0 resection rates were already high. The combination of ipilimumab and nivolumab with chemotherapy showed better efficacy compared with single‐agent immunotherapy, which is of considerable importance (Figure 4).

### 2.5 Safety of Neoadjuvant Immunotherapy Drugs in Network Meta‐Analysis

In the analysis of TRAEs, most RRs for immunotherapy compared with chemotherapy were close to 1, indicating a similar overall incidence of adverse events. For ipilimumab plus nivolumab (RR 1.08 [0.95, 1.22]), the RR value was slightly higher, indicating a possible increased risk of mild to moderate adverse events. However, for severe adverse events (TRAEs ≥ Grade 3), ipilimumab plus nivolumab showed a relatively lower risk compared with chemotherapy (RR 0.47 [0.17, 1.36]). These findings suggest that, in terms of adverse event control, adding immunotherapy agents is comparable to neoadjuvant chemotherapy alone (Figure 4).

### 2.6 Stratified Analysis for pCR

**FIGURE 5:**
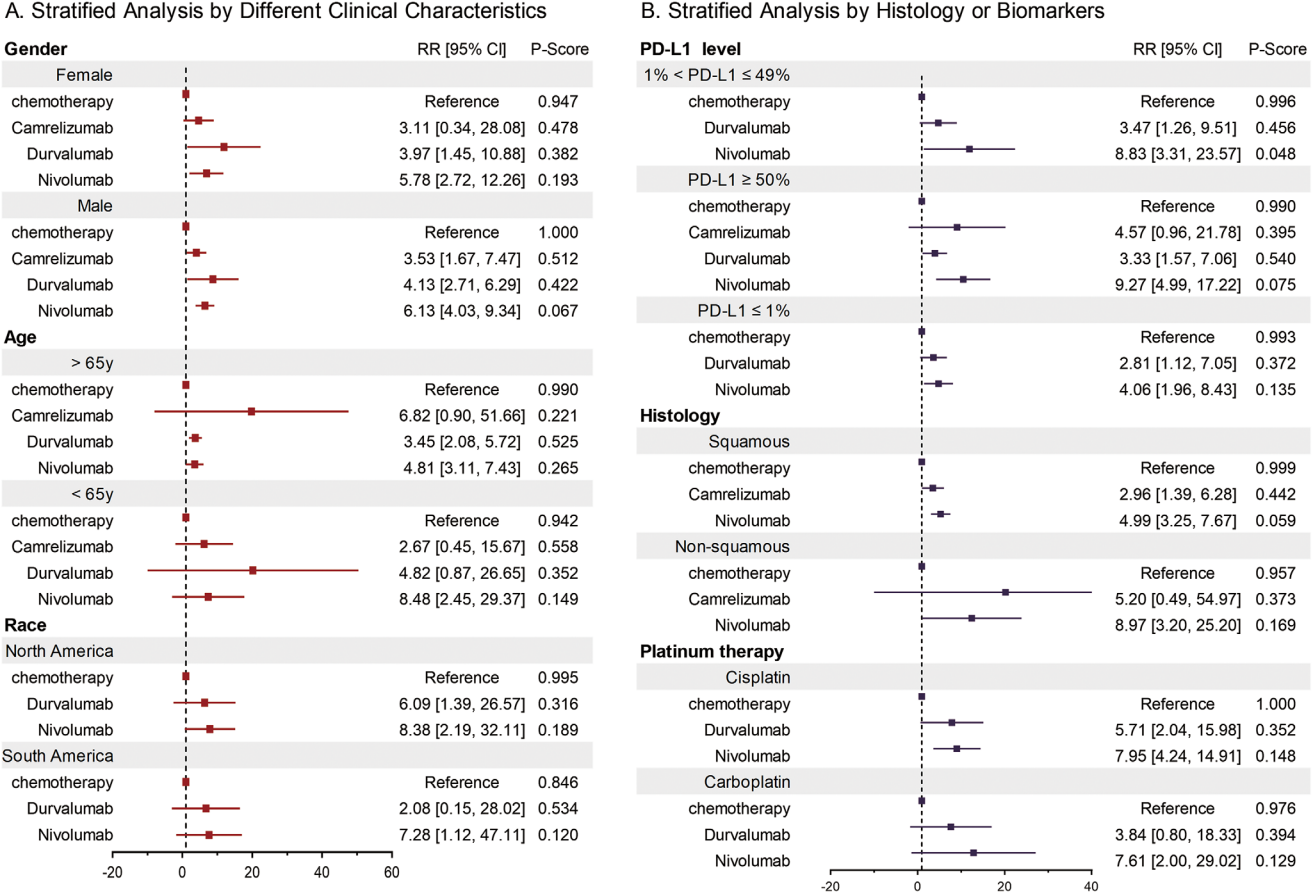
Stratified analysis of neoadjuvant immunotherapy plus chemotherapy compared with neoadjuvant chemotherapy in network meta‐analysis. (A) Stratified analysis by clinical characteristics; (B) stratified analysis by histological or molecular biomarkers.

We analyzed the pCR outcomes for male and female patients separately. The results indicate that both male and female patients derive clinical benefits from immunotherapy, with male patients showing a slightly higher RR for nivolumab (RR 6.13 [4.03, 9.34]) compared with female patients (RR 5.78 [2.72, 12.26]). These findings suggest potential differences in treatment responses by sex. Furthermore, the analysis divided patients into two groups: those older than 65 years and those younger than 65 years. Younger patients demonstrated higher RRs for nivolumab (RR 8.48 [2.45, 29.37]) compared with older patients (RR 4.81 [3.11, 7.43]), highlighting the influence of age on treatment efficacy. In terms of race, we performed analyses for North American and South American subpopulations. For example, in North America, nivolumab showed an RR of 8.38 [2.19, 32.11], suggesting a robust benefit in this subgroup. Similarly, significant benefits were observed in South America. PD‐L1 expression levels are a well‐established biomarker for predicting the efficacy of immune checkpoint inhibitors. We found that patients with PD‐L1 ≥50% exhibited superior efficacy for nivolumab (RR 9.27 [4.99, 17.22]) compared with those with PD‐L1 levels of 1–49% (RR 8.83 [3.31, 23.57]) or ≤1% (RR 4.06 [1.96, 8.43]). This trend aligns with the mechanism of action of PD‐1/PD‐L1 inhibitors, which rely on PD‐L1 expression for T‐cell activation. The results confirm that PD‐L1 levels are a crucial determinant of response to immunotherapy and should be considered when selecting patients for neoadjuvant immunotherapy. Meanwhile, stratified analyses by histological type revealed that nonsquamous NSCLC patients had a higher RR for nivolumab (RR 8.97 [3.20, 25.20]) compared with squamous NSCLC patients (RR 4.99 [3.25, 7.67]). These findings highlight the importance of histological subtypes in tailoring treatment strategies. We also examined the efficacy of nivolumab in cisplatin‐ and carboplatin‐based regimens. nivolumab demonstrated consistent efficacy across both regimens, with a slightly higher RR in the cisplatin group (RR 7.95 [2.44, 19.41]) (Figure 5).

### 2.7 Risk of Bias, Inconsistency, Publication Bias, and Quality Assessment

In the nine global RCTs, none of the studies exhibited a high risk of bias across seven domains (Table S3). The analysis of trial protocols and detailed reporting of progress on ClinicalTrials.gov bolstered our confidence in the low risk of bias results for this study. Funnel plots and Egger's test indicated no publication bias in outcomes other than surgery rate. However, it is noteworthy that after conducting a trim‐and‐fill analysis to account for potential publication bias, the adjusted RR was 1.04 [1.00, 1.07), which was not a significant result (*p* = 0.07) (Figures S8–S22). The global consistency results also did not show any relevant inconsistency (Table S1).

The Grading of Recommendations, Assessment, Development and Evaluation (GRADE) quality assessment of the safety and efficacy of neoadjuvant immunotherapy plus chemotherapy compared with neoadjuvant chemotherapy rated the evidence as moderate to high. Due to the small number of cases, MPR was downgraded, but its high effect size was upgraded by one grade. The safety outcome was downgraded because of the statistical significance of its effect size. We considered the other results to be high‐grade recommendable evidence (Table S4). When assessing the results using the Evidence Class, the pooled meta‐analysis outcomes for EFS, pCR, and MPR of neoadjuvant immunotherapy plus chemotherapy were rated as Class IV evidence, primarily due to the case numbers being less than 1000. However, the statistical significance of results (*p* value < 10⁻⁶), the absence of evidence indicating small study effects and excessive significance bias, the 95% prediction interval excluding null values, and the absence of significant heterogeneity suggest that these outcomes may potentially reach high‐level evidence with more clinical trials. The surgery rate, R0 resection and TRAEs ≥ Grade 3 were rated as Class IV evidence. Due to statistical nonsignificance, all TRAEs were rated as NS level. Regarding the network meta‐analysis results, the lack of head‐to‐head comparisons of neoadjuvant immunotherapy drugs and the statistical weakness of current results means that the recommendations and grading based on these results are minimal, and thus no recommendations or ratings are made.

## 3 Discussion

Since its clinical application, immunotherapy has rapidly become a widely used and highly effective treatment method. In particular, immunotherapy has been proven safe and effective for lung cancer's adjuvant and neoadjuvant treatment. Despite numerous recent RCTs comparing neoadjuvant immunotherapy plus chemotherapy with chemotherapy alone, higher levels of evidence are needed to substantiate their safety and efficacy. We conducted a pooled meta‐analysis and a network meta‐analysis on the safety and efficacy of neoadjuvant immunotherapy plus chemotherapy versus neoadjuvant chemotherapy, as well as the different effects of various neoadjuvant immunotherapy drugs.

Our findings on the safety and efficacy of neoadjuvant immunotherapy plus chemotherapy for lung cancer indicate that the addition of neoadjuvant immunotherapy offers significant benefits over chemotherapy alone. This is evidenced by the HR for EFS of 0.57 [0.50, 0.66] and about four times increase in pCR and MPR pathological benefits (RRs of 4.17 [3.07, 5.66] and 3.57 [2.51, 5.10], respectively), highlighting the advantages of the immunotherapy plus chemotherapy neoadjuvant treatment. This combination significantly enhances treatment outcomes and patient survival. The heterogeneity of several results was low, with no significant bias detected, and both the methodological assessment by AMSTAR2 and the GRADE scoring were of the highest level. It is noteworthy that the Evidence Class rated EFS, pCR, and MPR as Class IV evidence, indicating weak evidence. The extremely small *p* values, along with no evidence of small study effects, excessive significance bias, and heterogeneity, suggest that the primary limitation preventing these studies from achieving a higher level of evidence (even Class I) is merely the insufficient number of participants. Therefore, these Class IV evidence outcomes should not be undervalued, as they possess the potential to be considered high‐level evidence in future assessments by Evidence Class. Based on this, we believe these outcomes strongly support the clinical practice of adding immunotherapy to chemotherapy as a neoadjuvant treatment.

Regarding the surgery rate (RR 1.04 [1.00, 1.08]) and R0 resection rate (RR 1.04 [1.01, 1.08]), the benefits of adding neoadjuvant immunotherapy seem modest. Clinically, the maximum relative risk reduction (RRR) is 8%, indicating a 8% increased likelihood of undergoing surgical resection and achieving R0 resection for patients receiving the combined neoadjuvant immunotherapy compared with those receiving neoadjuvant chemotherapy alone. Even the minimal RRR suggests a 1% possibility of achieving R0 resection in NSCLC patients. Considering the 31% difference in 5‐year survival rates between complete and incomplete resection in lung cancer patients, there's reason to believe that adding neoadjuvant immunotherapy could provide greater benefits for subsequent surgery compared with neoadjuvant chemotherapy alone [20]. However, it is essential to note that some publication bias was observed for the surgery rate outcome, which, despite slight changes after trim‐and‐fill analysis, requires cautious interpretation. Regarding safety, all TRAEs was statistically significant, but from a clinical aspect, neoadjuvant immunotherapy plus chemotherapy does lead to a slightly higher incidence of adverse events, especially in higher grade TRAEs (RR 1.11 [1.03, 1.19]), and there is a need to pay more attention to adverse events in patients.

In the network meta‐analysis, it is crucial to note that the connections in the network diagram primarily focus on comparisons with neoadjuvant chemotherapy, with very few comparisons among the different neoadjuvant immunotherapy drugs. The results of the network meta‐analysis, which include both direct and indirect comparisons, necessitate cautious interpretation due to the lack of direct comparisons. However, the findings still clarify that adding neoadjuvant immunotherapy offers better survival benefits and surgical outcomes for lung cancer patients, supporting the combined use of neoadjuvant immunotherapy and chemotherapy. The safety outcomes are similar to those of the pooled meta‐analysis. Of particular interest is the indirect comparison analysis, which shows that neoadjuvant chemotherapy combining ipilimumab and nivolumab, two immunotherapy drugs, results in better EFS, pCR, and MPR than neoadjuvant therapy with a single immunotherapy drug plus chemotherapy. While the incidence of all TRAEs is similar to that of adding a single immunotherapy drug, the occurrence of high‐grade TRAEs significantly decreases. Although more research is needed to confirm this conclusion, it points to a potentially more effective pathway: neoadjuvant therapy with dual immunotherapy drugs plus chemotherapy. We also compared the performance of different immunotherapy drugs in the neoadjuvant setting, with similar types of immunotherapy drugs showing better survival benefits. It should be perceived that due to the limited number of clinical studies, our comparison results can only preliminarily demonstrate the differences among the drugs, and robust evidence from more clinical studies is required to substantiate these findings.

In the stratified analysis, we found that age, gender, and race all had varying impacts on pCR. Similarly, with the use of immunotherapy, the results confirmed that PD‐L1 levels are a crucial determinant of the response to immunotherapy and should be taken into account when selecting patients for neoadjuvant immunotherapy. Importantly, stratified analysis by histological type showed that nonsquamous NSCLC patients had a RR for nivolumab (8.97 [3.20, 25.20]) compared with squamous NSCLC patients (4.99 [3.25, 7.67]). Nonsquamous NSCLC is often characterized by a higher mutational burden, which has been linked to enhanced immunogenicity and a better response to immune checkpoint inhibitors [21]. Additionally, the efficacy of nivolumab in cisplatin‐ and carboplatin‐based regimens demonstrated consistent outcomes across both treatments, with a slightly higher HR observed in the cisplatin group (7.95 [2.44, 19.41]). This may be attributed to the stronger immunogenic effects of cisplatin compared with carboplatin, which can enhance the response to immunotherapy by promoting antigen presentation and immune activation [22, 23].

Our study has certain limitations. The limited number of included studies led to a sparse network structure and instability in the transitivity results, making it challenging to accurately compare the efficacy of different immunotherapy agents as neoadjuvant treatments. However, the primary goal of our study was to explore the overall benefit of adding immunotherapy to neoadjuvant chemotherapy, and our results consistently demonstrated significant efficacy and clinical benefit.

## 4 Conclusion

Neoadjuvant immunotherapy plus chemotherapy, compared with neoadjuvant chemotherapy, provides NSCLC patients with greater survival benefits in terms of EFS, pathological outcomes, surgery rates, and R0 resection rates, albeit with a slight increase in TRAEs, especially Grade ≥ 3. Among these, neoadjuvant therapy that combines two immunotherapy drugs with chemotherapy has performed better results, yet further clinical research is required for validation. Hence, the combination of neoadjuvant immunotherapy and chemotherapy should receive wider attention and application in the treatment of lung cancer, offering new hope for treatment to lung cancer patients. Future studies should continue to explore the efficacy and safety of different immunotherapy drugs to refine and personalize treatment plans, further improving survival rates and the quality of life for lung cancer patients.

## 5 Methods

### 5.1 Search Strategy

This study was reported in strict accordance with the PRISMA guidelines, registered with PROSPERO, CRD42024519654. Searches were conducted in PubMed, Embase, and the Cochrane Library databases from their inception until November 22, 2024, with additional searches completed through the global oncology conferences, and ClinicalTrials.gov without any language restrictions.

### 5.2 Study Selection

Two reviewers (L. S. and M. Y. L.) worked independently using the Rayyan software system to screen abstracts and assess the full texts of eligible studies, with any discrepancies resolved through consensus by a third reviewer (Y. J. Q.). The studies included met the following criteria: (1) The study was a phase II or III randomized controlled trial; (2) all participants were pathologically diagnosed with NSCLC; (3) the experimental group received neoadjuvant treatment with a combination of immunotherapy and chemotherapy, while the control group received a combination of immunotherapy and chemotherapy or only chemotherapy as neoadjuvant treatment; (4) the study accurately and completely reported the outcomes.

### 5.3 Data Extraction

For each eligible study, two reviewers (L. S. and M. Y. L.) independently extracted the following information: basic characteristics of the study (publication year, type of clinical study), population (sample size, sociodemographic characteristics of the patients, clinical characteristics of the patients), intervention measures (category of drugs and dosages), and outcomes. This meta‐analysis used EFS as the primary outcome, as specified in randomized controlled trials. Other outcomes included pCR, MPR, surgery rate, R0 resection rate, all TRAEs, and TRAEs ≥ Grade 3. Any questions and disagreements were resolved through consensus by a third reviewer (L. H.).

### 5.4 Data Analysis

This study initially conducted a pooled meta‐analysis to verify the safety and efficacy of neoadjuvant immunotherapy plus chemotherapy compared with neoadjuvant chemotherapy. For network meta‐analysis, the frequentist approach is more straightforward in terms of model specification and interpretation, enhancing transparency and reproducibility in the analysis. The network meta‐analysis was conducted using the “netmeta” package in R software (version 4.3.3). This method allows for the simultaneous comparison of multiple interventions, synthesizing direct and indirect evidence to estimate relative effects between interventions and establish rankings. The chemotherapy alone group was used as the reference control group, and forest plots were constructed to display the effects of different interventions visually. A network plot was created to depict the relationships within the network, where each node represents a treatment, and the edges between nodes represent the comparisons between treatments. All analyses estimated effect sizes using RRs or HRs and 95% confidence intervals (CIs). To assess the relative effectiveness of the treatments, P‐scores were calculated for each, ranging from 1 (worst) to 0 (best), reflecting their relative effectiveness within the network. Publication bias was estimated through visual inspection of funnel plots and Egger's test. If the funnel plot showed significant asymmetry and Egger's test *p* value was less than 0.05, trim‐and‐fill analysis was conducted to further assess whether the presence of publication bias could affect the results. Sensitivity analyses used the cumulative approach to determine the effect of gradually adding research findings on the results. Due to significant inconsistencies and incompleteness in the subgroup‐specific HRs/RRs and corresponding 95% CIs for EFS and MPR across the original studies, reliable and rigorous stratified subgroup analyses were unfortunately not feasible. Consequently, detailed subgroup analyses were restricted exclusively to pCR, for which sufficient data were available.

### 5.5 Risk of Bias Assessment

Two independent reviewers (L. S. and D. J. X.) used the Cochrane Collaboration's risk of bias assessment tool to evaluate the risk of bias in the included studies, assessing biases in random sequence generation, allocation concealment, blinding, incomplete outcome data, selective reporting, and other potential biases. Each domain was judged as having a low, unclear, or high risk of bias. A study was deemed to have a low risk of bias if all assessed domains were judged to be at low risk. The study was considered to have an unclear risk of bias if any domain was assessed as unclear and a high risk of bias was attributed when any item was at high risk.

### 5.6 Evidence Quality Assessment

The quality of the evidence was assessed using the GRADE approach, taking into account the risk of bias, inconsistency, imprecision, indirectness, and publication bias. Based on these factors, evidence quality was categorized as high, moderate, low, or very low [24]. The quality of network estimates is rated lower in the presence of evidence showing inconsistency between direct and indirect estimates. Moreover, outcomes were also evaluated according to the Evidence Classification standards, focusing mainly on the statistical quality of outcome indicators [25, 26, 27]. Evidence is categorized into four levels: Class I (convincing evidence), Class II (highly suggestive evidence), Class III (suggestive evidence), Class IV (weak evidence), and NS (nonsignificant). The Appendix, Supporting Information provides detailed criteria for the Evidence Class.

## Author Contributions

Study concept and design: M. X. L., L. S., and L. H. Search strategy: M. Y. L., L. S., and L. Y. Y. Selection criteria: M. Y. L., L. S., and Y. J. Q. Quality assessment: L. S., Y. J. Q., and D. J. X. Drafting of the manuscript: L. S., M. Y. L., Z. F., M. X. L., and L. H. All authors read and approved the final manuscript.

## Ethics Statement

The authors have nothing to report.

## Conflicts of Interest

All authors declare no conflicts of interest.

## Supporting information



Supporting Information

## Data Availability

To ensure transparency and reproducibility of the study, the study data and Supporting Information will be available in the appendix upon request from the corresponding author. For more detailed data inquiries, researchers are encouraged to contact the corresponding author via email. Please note that data sharing is intended for academic research purposes only and not for other purposes.
