# Supplementary material for: Efficacy and Safety of Neoadjuvant Immunotherapy plus Chemotherapy versus Neoadjuvant Chemotherapy in Non‐Small Cell Lung Cancer Treatment: A Mixed Method Meta‐Analysis Based on Global Randomized Controlled Trials
Source: MedComm (2020). 2025 May 31;6(6):e70211. doi: 10.1002/mco2.70211 (PMC12126597; doi:10.1002/mco2.70211)
Supplement: Supplementary file 1 — Supporting Information [file MCO2-6-e70211-s001.docx]

**Supplementary appendix**

**Supplement to:**

**Efficacy and Safety of Neoadjuvant Immunotherapy Plus Chemotherapy vs. Neoadjuvant Chemotherapy in Non-small Cell Lung Cancer Treatment: A Mixed Method Meta-Analysis Based on Global Randomized Controlled Trials**

**Contents:**

**Search Strategy**

**Evidence Classification Criteria**

**Tables S1-S4**

**Figures S1-S22**

**The search strategy (Update to Nov 22, 2024)**

**PubMed**

((Neoadjuvant AND ((Therapy OR Therapies) OR Treatment)) OR Neoadjuvant OR neoadjuvant immunotherapy OR neoadjuvant chemoimmunotherapy OR neoadjuvant immune checkpoint inhibitor) AND (lung cancer OR lung carcinoma OR NSCLC OR non-small cell lung cancer) AND (clinical trial [Filter]).

**Embase**

(('Neoadjuvant':af AND (('Therapy':af OR 'Therapies':af) OR 'Treatment':af)) OR 'Neoadjuvant':af OR 'neoadjuvant immunotherapy':af OR 'neoadjuvant chemoimmunotherapy':af OR 'neoadjuvant immune checkpoint inhibitor':af) AND ('lung cancer':af OR 'lung carcinoma':af OR 'NSCLC':af OR 'non-small cell lung cancer':af) AND 'clinical trial':af.

**Cochrane**

(("Neoadjuvant" AND (("Therapy" OR "Therapies") OR "Treatment")) OR "Neoadjuvant" OR "neoadjuvant immunotherapy" OR "neoadjuvant chemoimmunotherapy" OR "neoadjuvant immune checkpoint inhibitor") AND ("lung cancer" OR "lung carcinoma" OR "NSCLC" OR "non-small cell lung cancer") AND "clinical trial"

**Evidence classification criteria [1]**

**Class I:**

Convincing evidence, >1000 cases (or >20,000 participants for continuous outcomes); statistical significance at P<10−6 (random effects); no evidence of small study effects and excess significance bias; 95% prediction interval excluded null value; no large heterogeneity (I2<50%).

**Class II:**

Highly suggestive evidence, >1000 cases (or > 20,000 participants for continuous

outcomes), statistical significance at P<10^−6^ (random effects), and largest study with 95% confidence interval excluding null value.

**Class III:**

Suggestive evidence, >1000 cases (or >20,000 participants for continuous outcomes), and statistical significance at P<0.001.

**Class IV:**

Weak evidence, Remaining significant associations with P<0.05.

**NS:**

Non-significant, P>0.05.

*[1] Huang Y, Chen Z, Chen B, Li J, Yuan X, Li J, Wang W, Dai T, Chen H, Wang Y, Wang R, Wang P, Guo J, Dong Q, Liu C, Wei Q, Cao D, Liu L. Dietary sugar consumption and health: umbrella review. BMJ. 2023 Apr 5;381:e071609. doi: 10.1136/bmj-2022-071609. PMID: 37019448; PMCID: PMC10074550.*

| Global Inconsistency |  |  |  |
| --- | --- | --- | --- |
| Outcome | Q | df | P |
| All TRAEs | 2.98502369 | 3 | 0.08403806 |
| MPR | 8.841E-29 | 1 | / |
| pCR | 0.95248397 | 4 | 0.62111317 |
| R0 resection | 0.09978198 | 2 | 0.75209143 |
| Surgery rate | 2.0242185 | 3 | 0.15480854 |
| TRAEs > Grade 3 | 2.8364979 | 2 | 0.09214484 |
| EFS | 0.93 | 4 | 0.9197 |
|  |  |  |  |
| Design Specific Inconsistency |  |  |  |
| Outcome | Design | Q | P |
| All TRAEs | chemotherapy:durvalumab | 1.269753 | 0.259813089 |
| MPR | chemotherapy:nivolumab | 0.085614 | 0.769829255 |
| pCR | chemotherapy:nivolumab | 0.95248397 | 0.6211131653 |
| R0 resection | chemotherapy:nivolumab | 0.146348 | 0.929439025 |
| Surgery rate | chemotherapy:durvalumab | 0.054488 | 0.815430145 |
| TRAEs > Grade 3 | chemotherapy:nivolumab | 2.836919 | 0.242086658 |
|  |  |  |  |
| Random Effects Inconsistency |  |  |  |
| Outcome | Q | df | Tau within |
| All TRAEs | 4.44E-16 | 0 | 0.05578539 |
| MPR | 0 | 0 | 0 |
| pCR | 0 | 0 | 0 |
| R0 resection | 0 | 0 | 0 |
| Surgery rate | 0 | 0 | 0 |
| TRAEs > Grade 3 | -2.2E-16 | 0 | 0.198687329405991 |
| EFS | 0 | 0 | 0 |

**Table S1. Inconsistency Test Results**

**Table S2. League Table.**

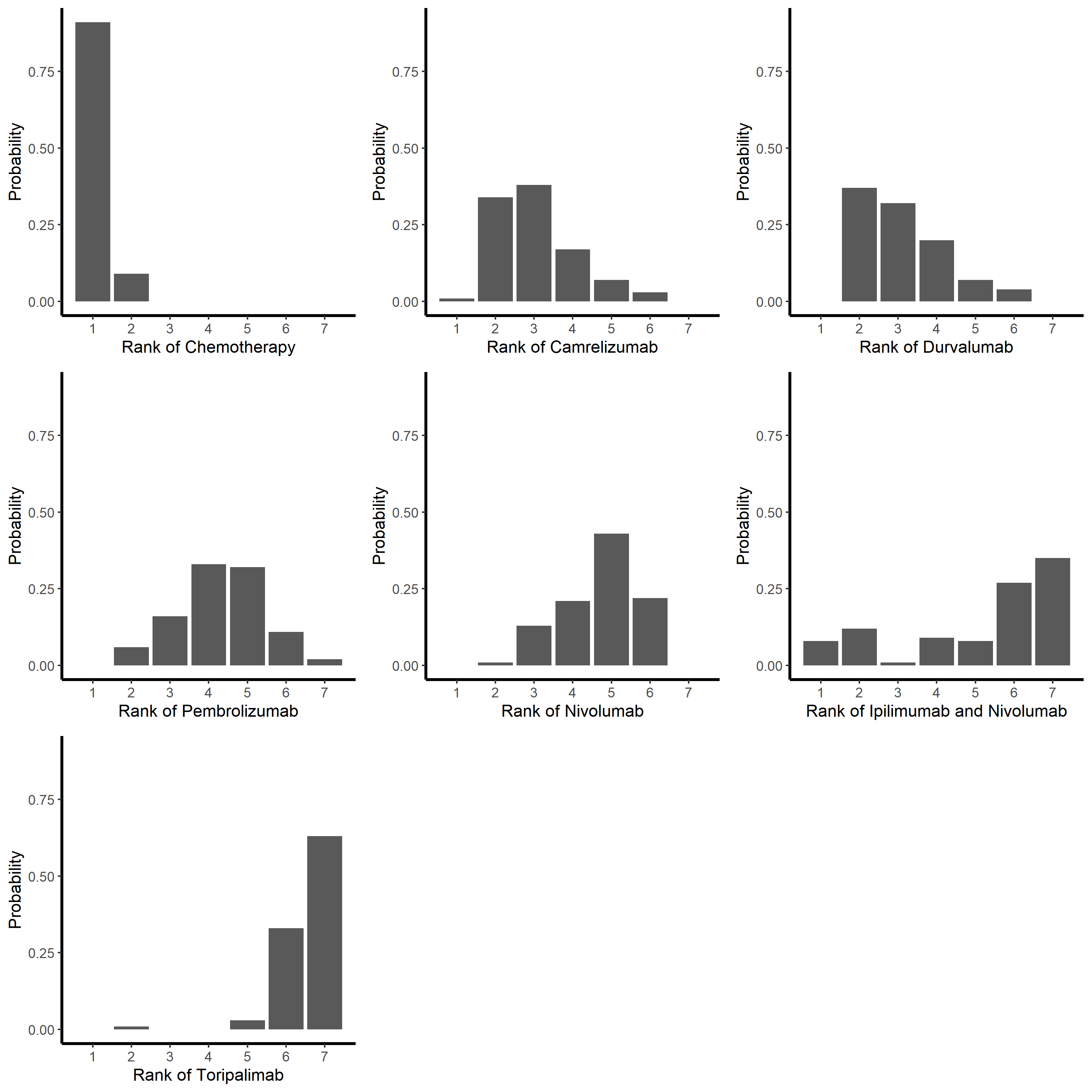


**Figure S1. Rankogram of EFS**


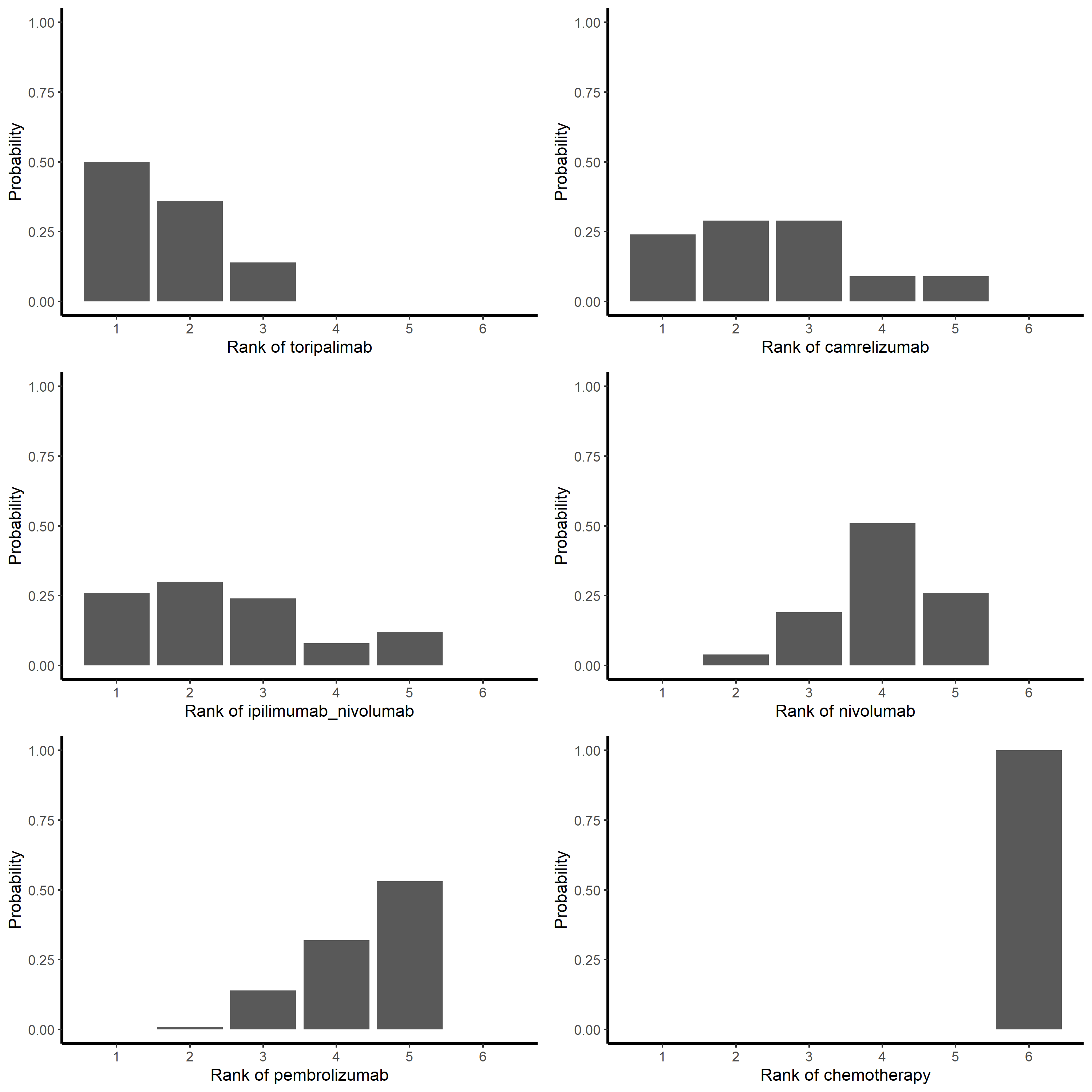


**Figure S2. Rankogram of MPR**
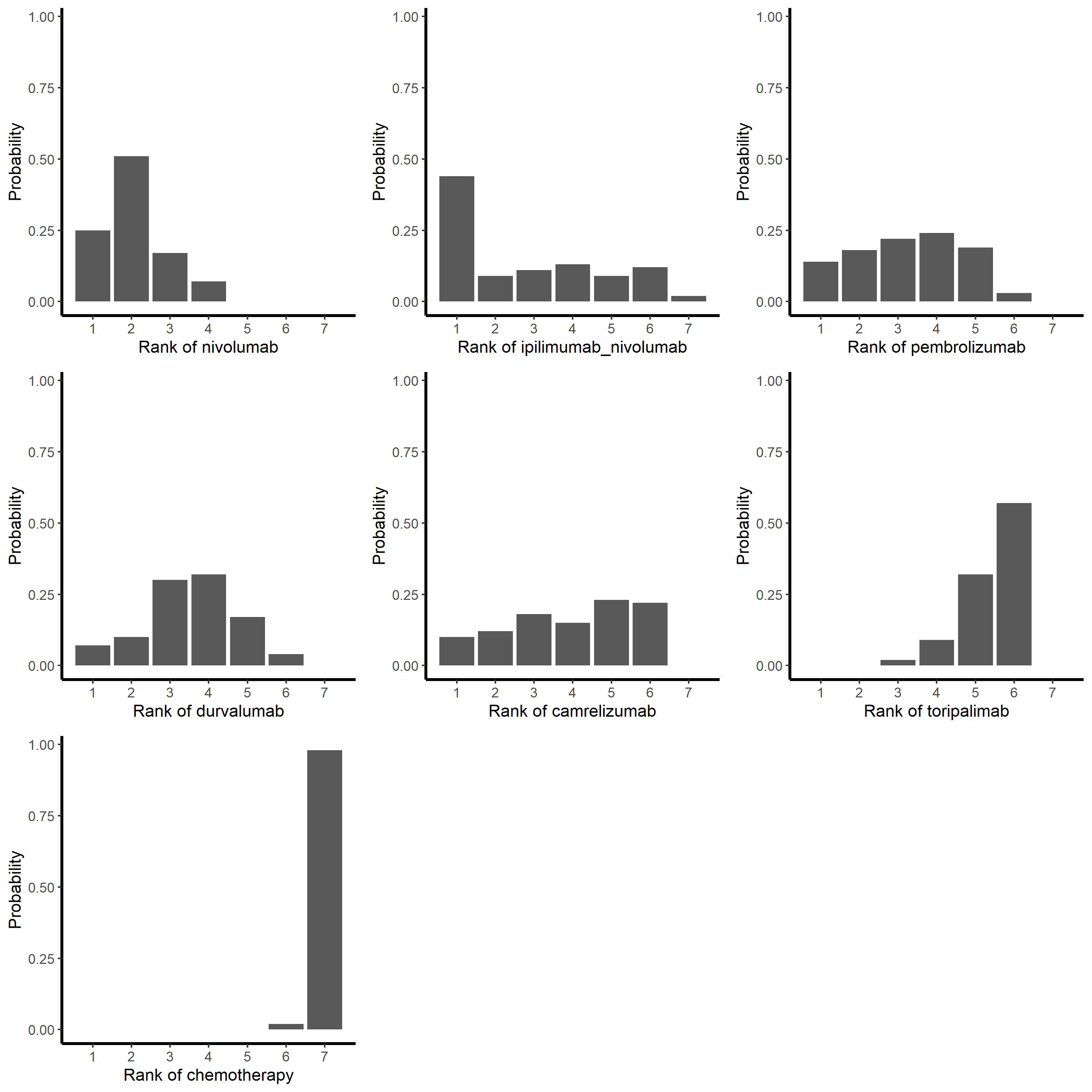


**Figure S3. Rankogram of pCR**


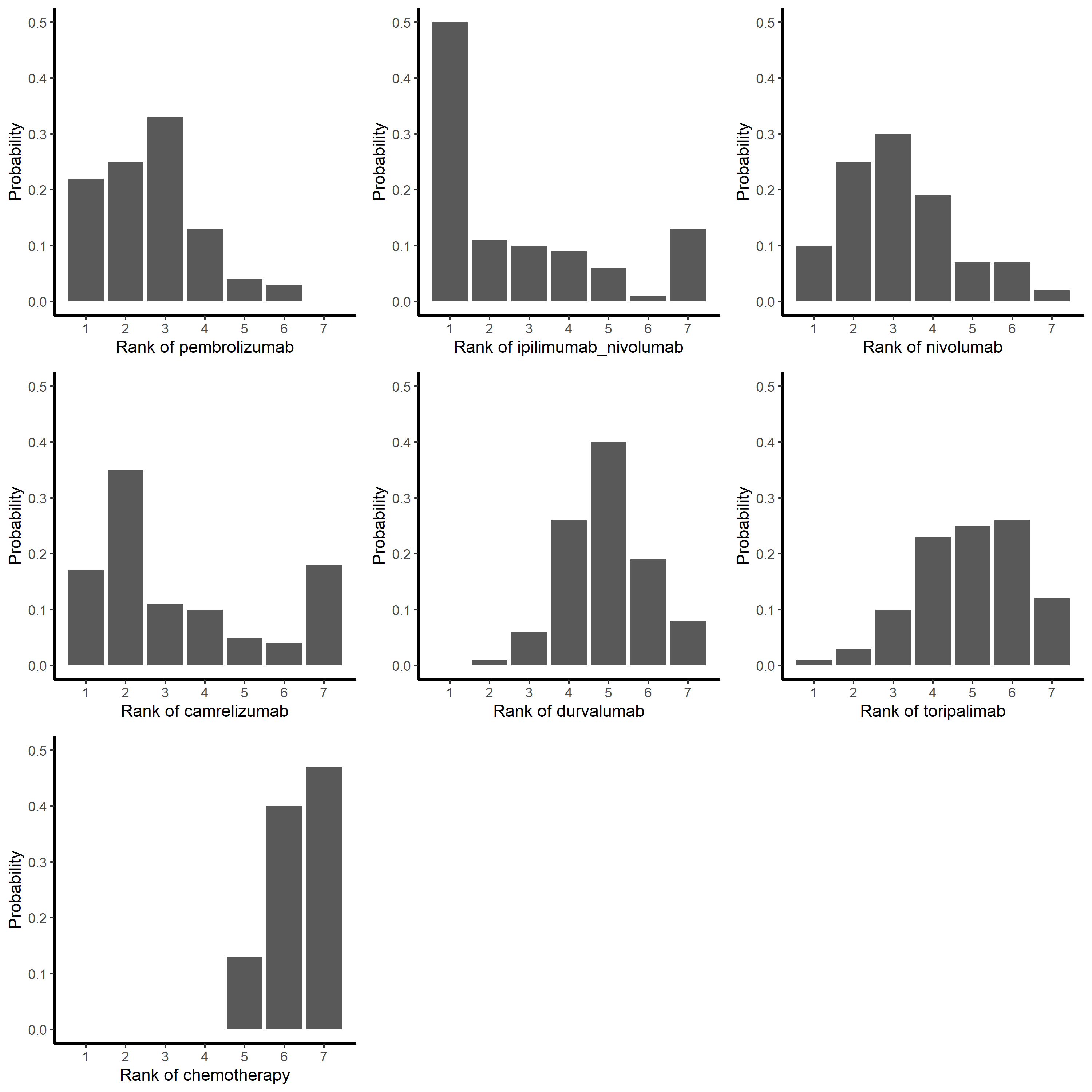


**Figure S4. Rankogram of R0 resection**


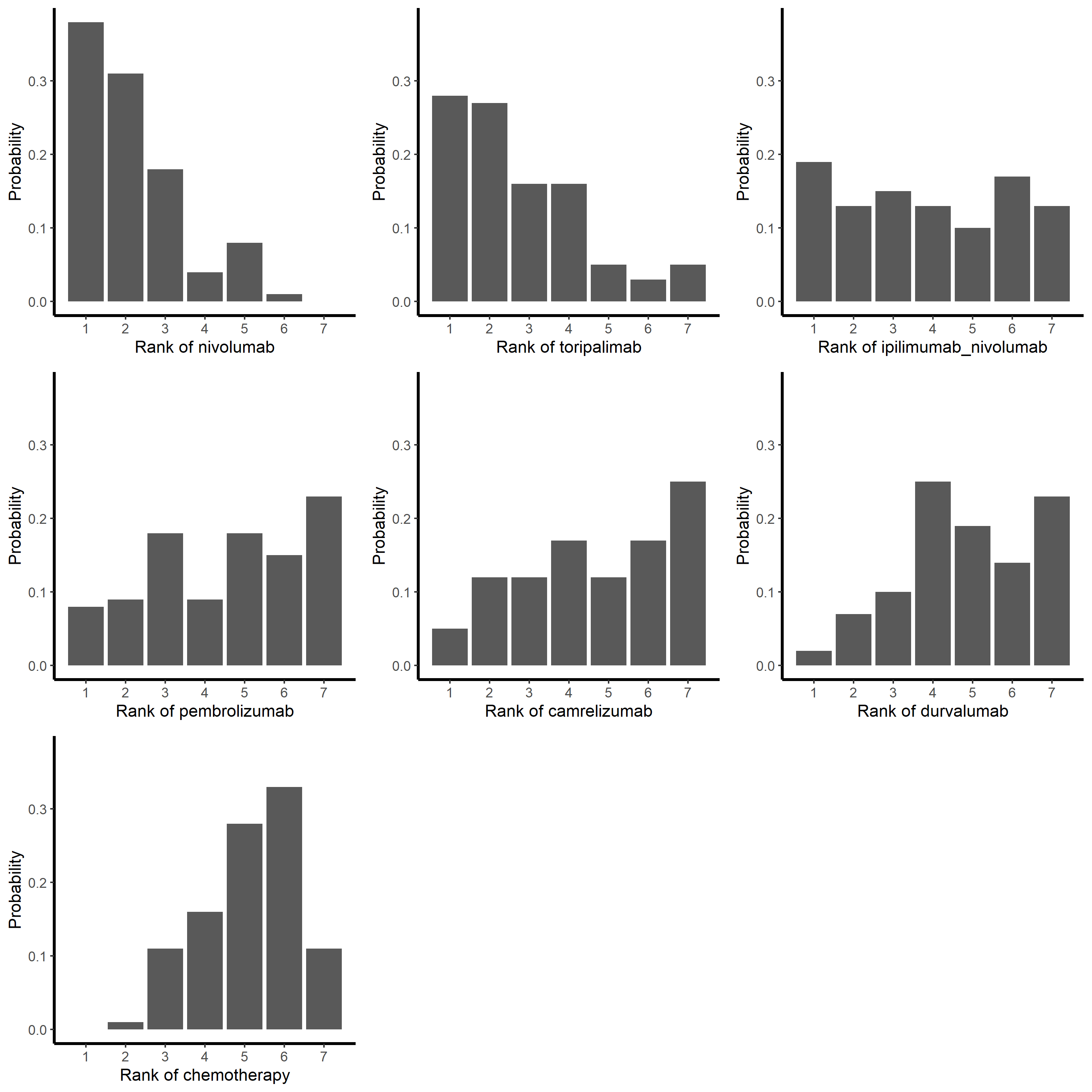


**Figure S5. Rankogram of Surgery rate**
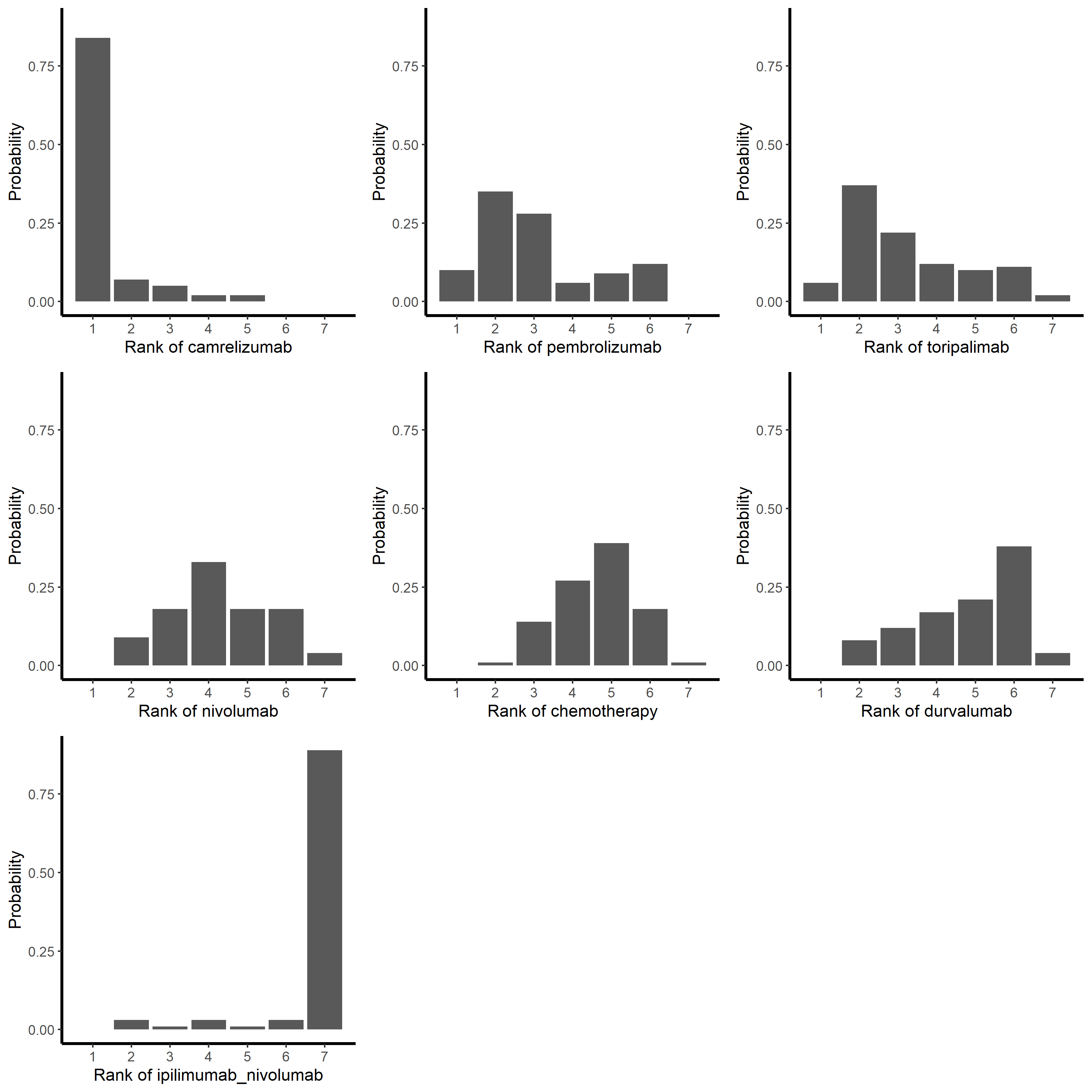


**Figure S6. Rankogram of TRAEs > Grade 3**


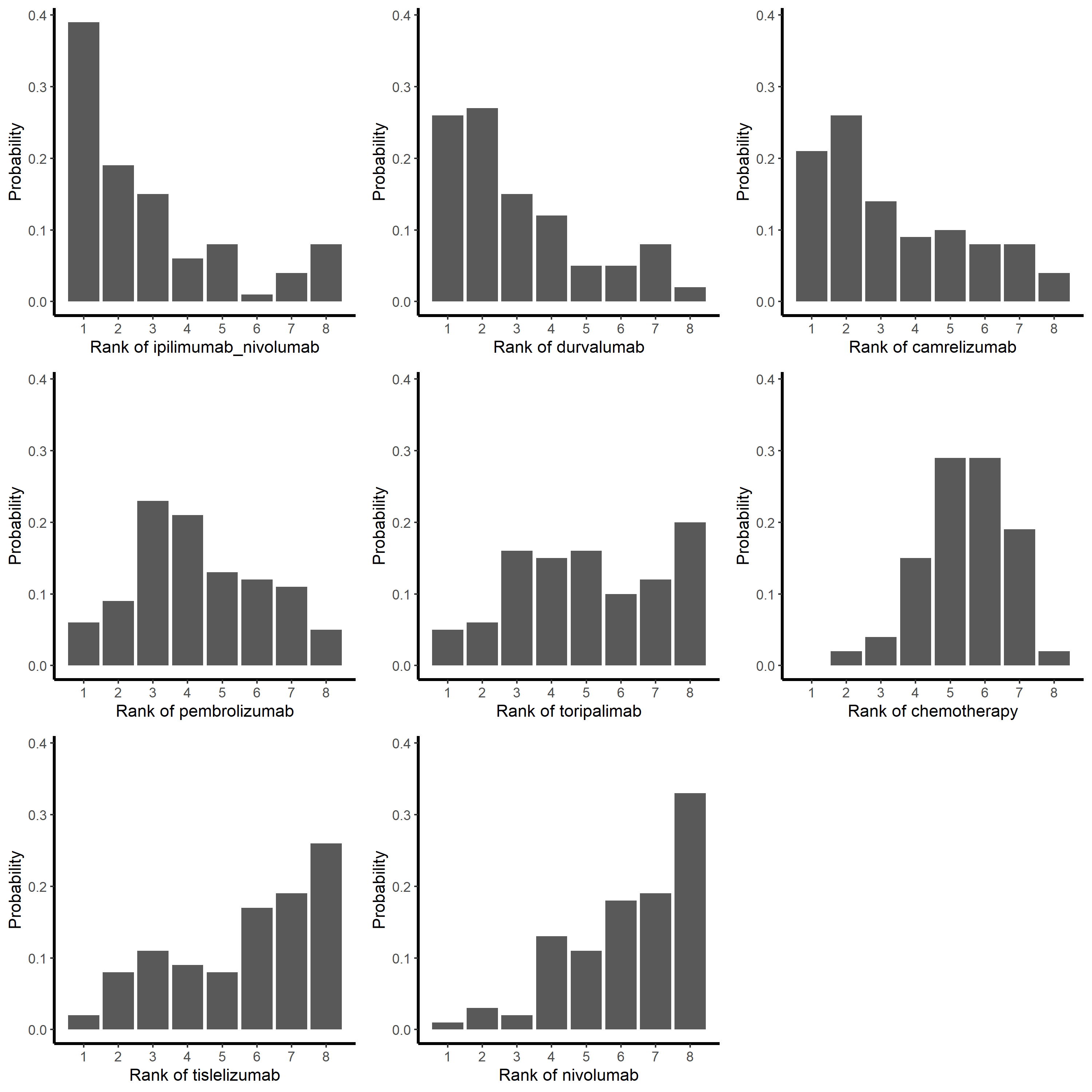


**Figure S7. Rankogram of All TRAEs**


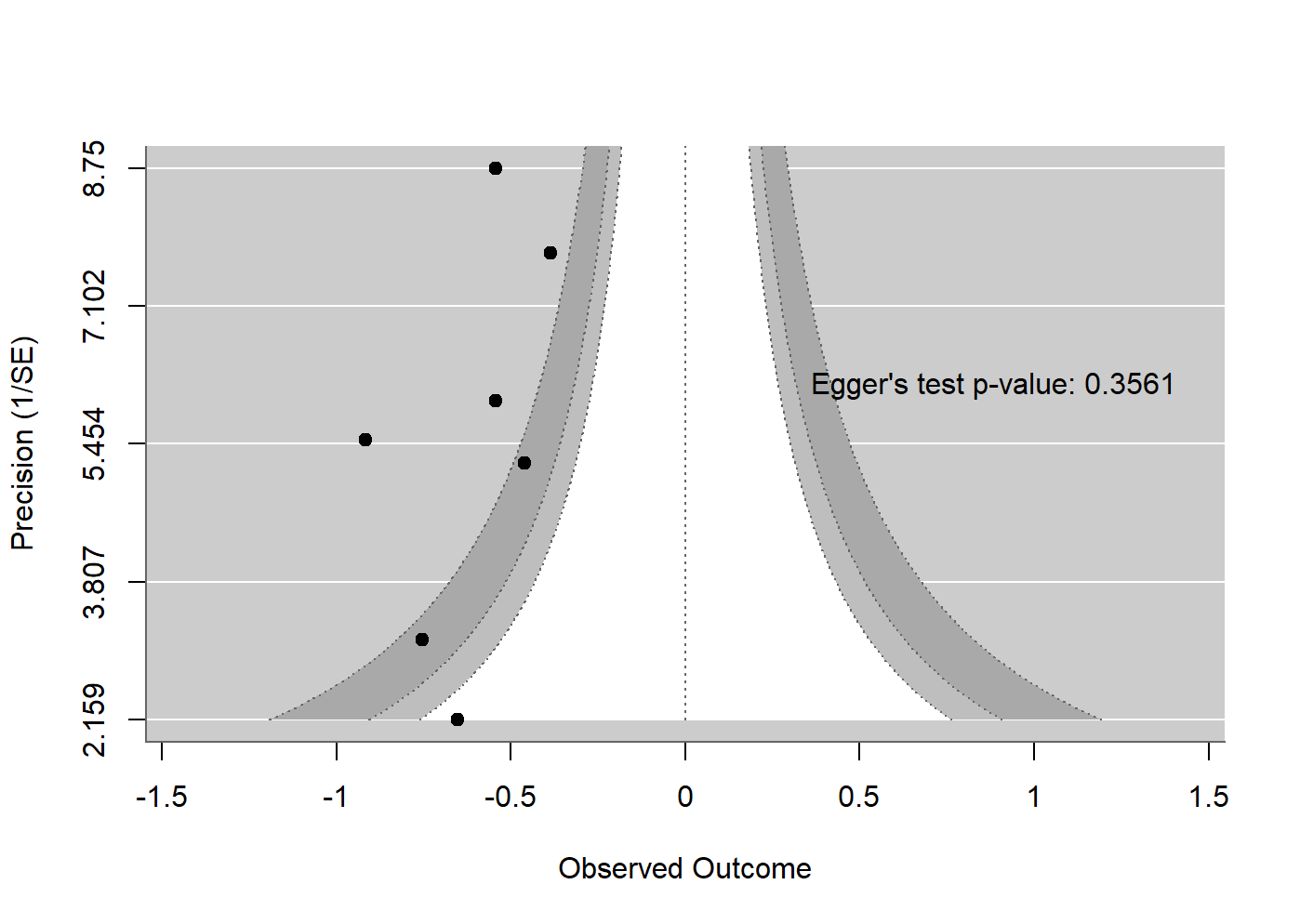


**Figure S8.** **Funnel plot and Egger’s regression of EFS.**


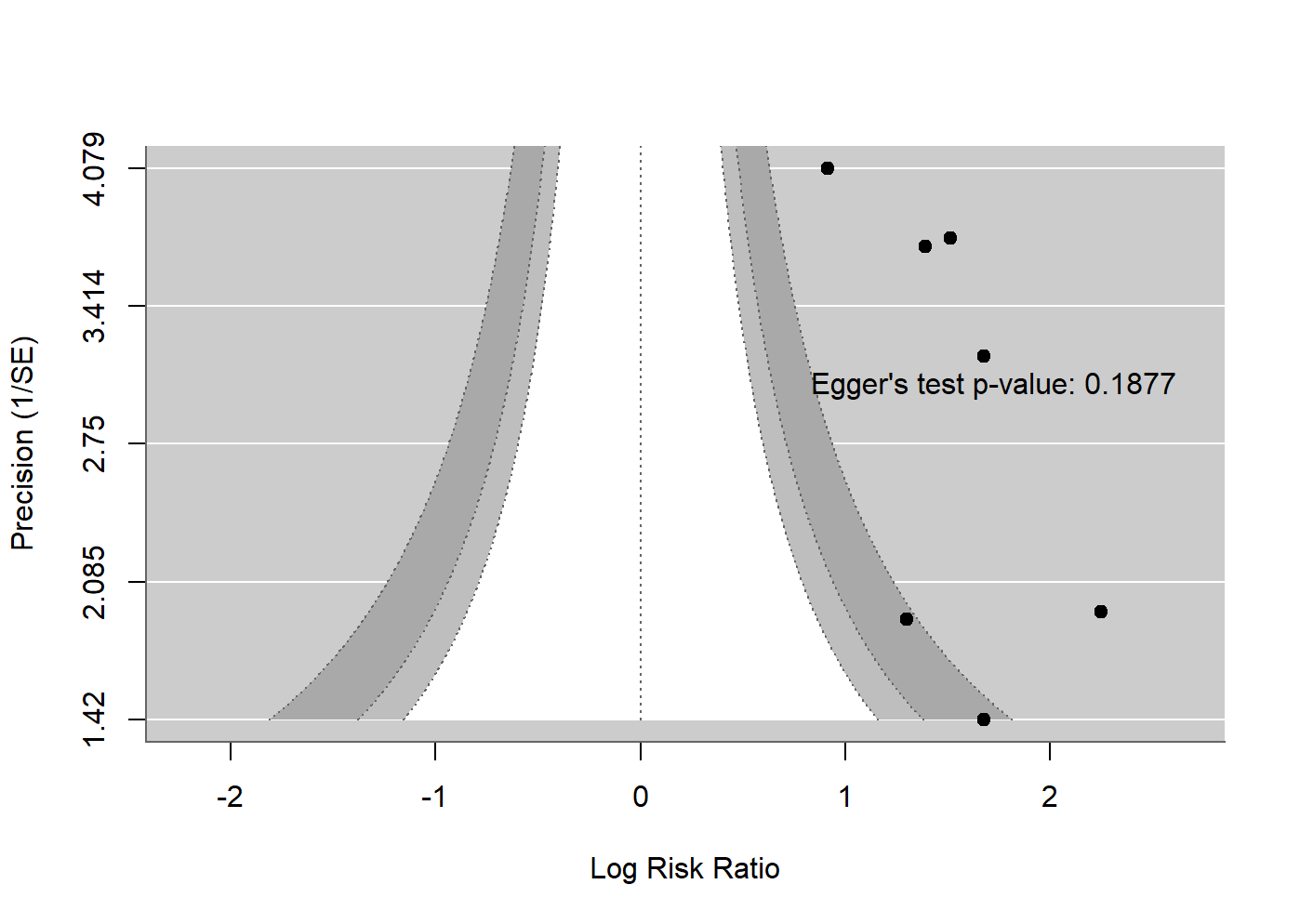


**Figure S9. Funnel plot and Egger’s regression of pCR.**


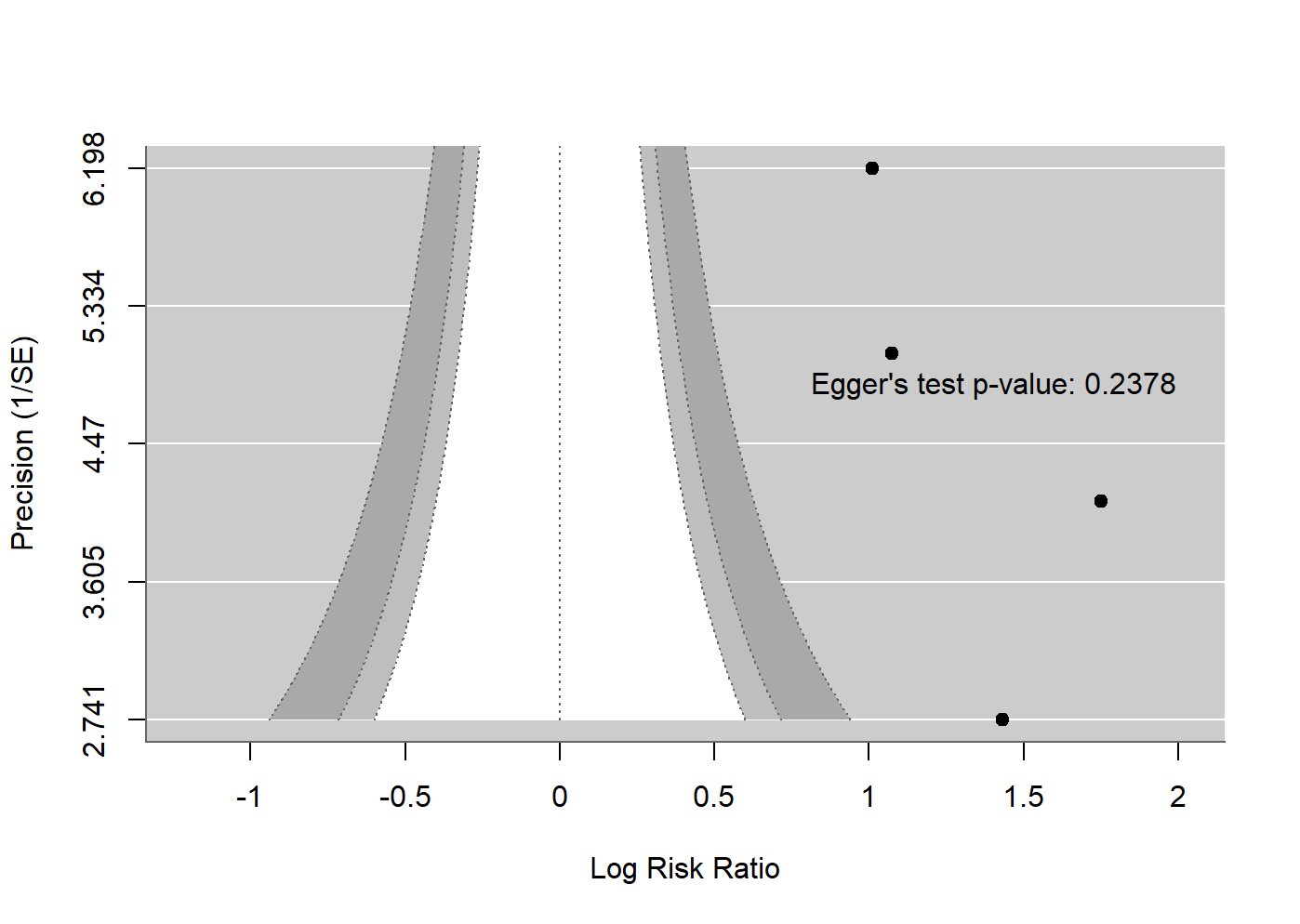


**Figure S10. Funnel plot and Egger’s regression of MPR.**

**
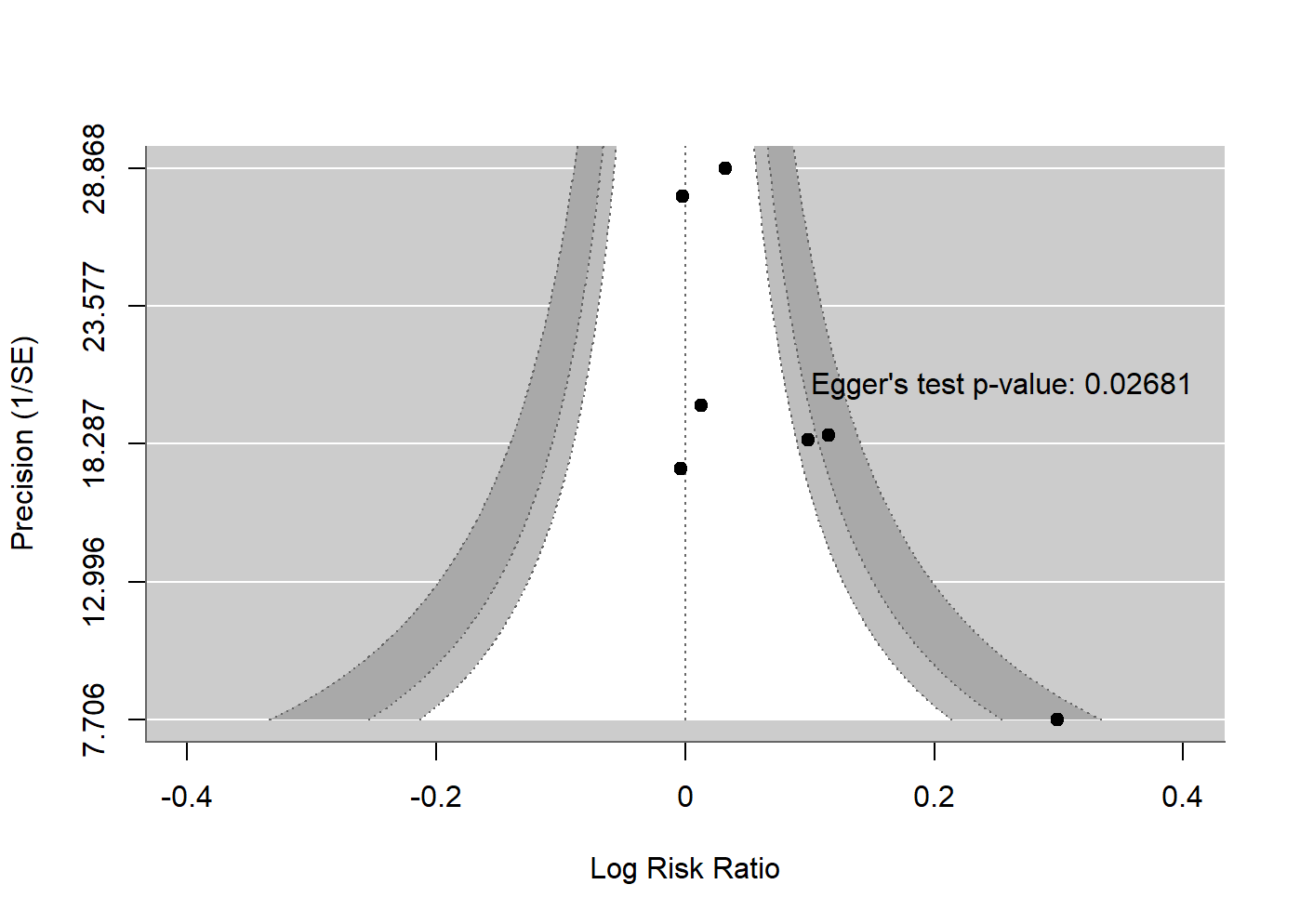
**

**Figure S11. Funnel plot and Egger’s regression of Surgery rate.**


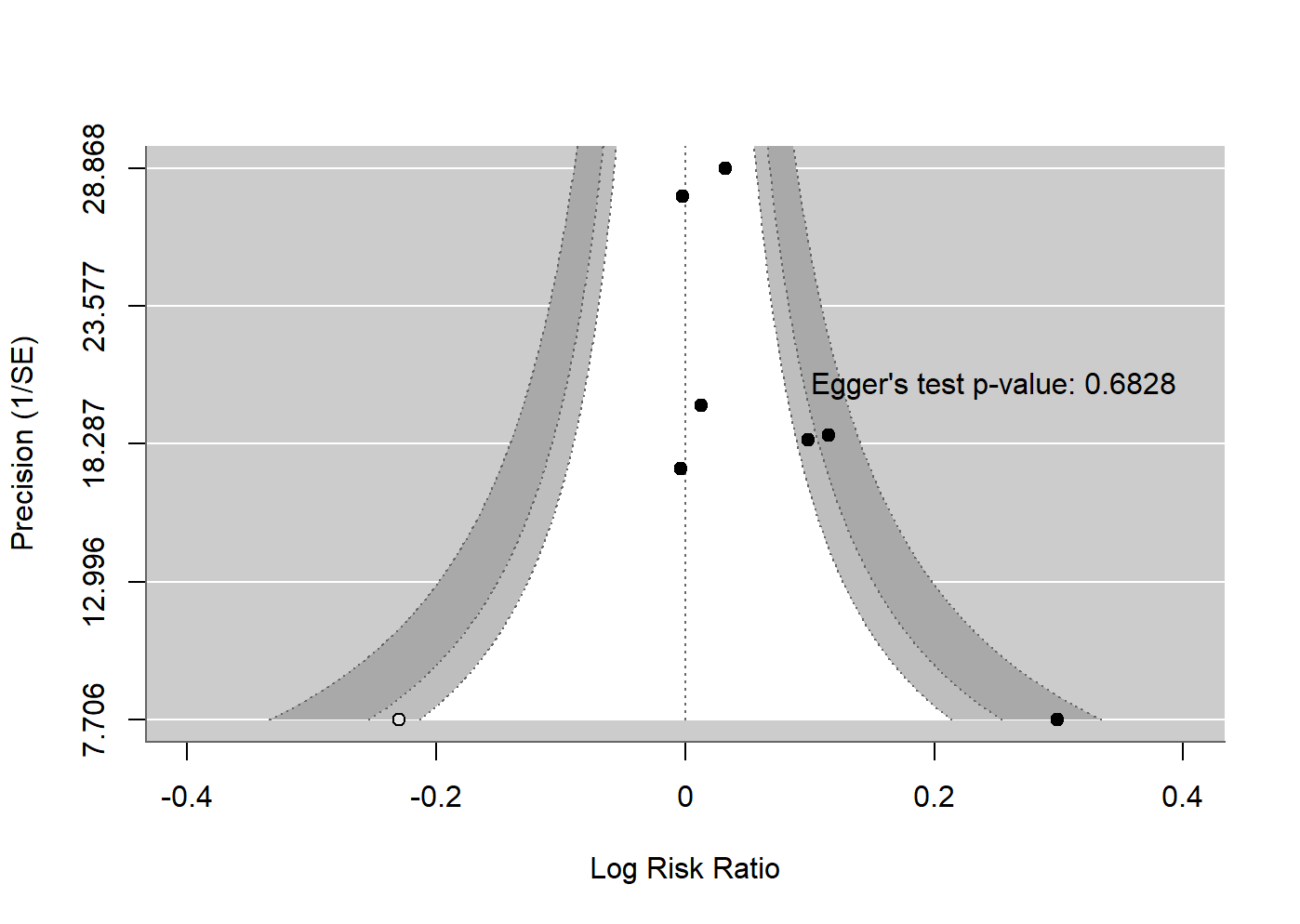


**Figure S12. Trimfill plot of Surgery rate.**


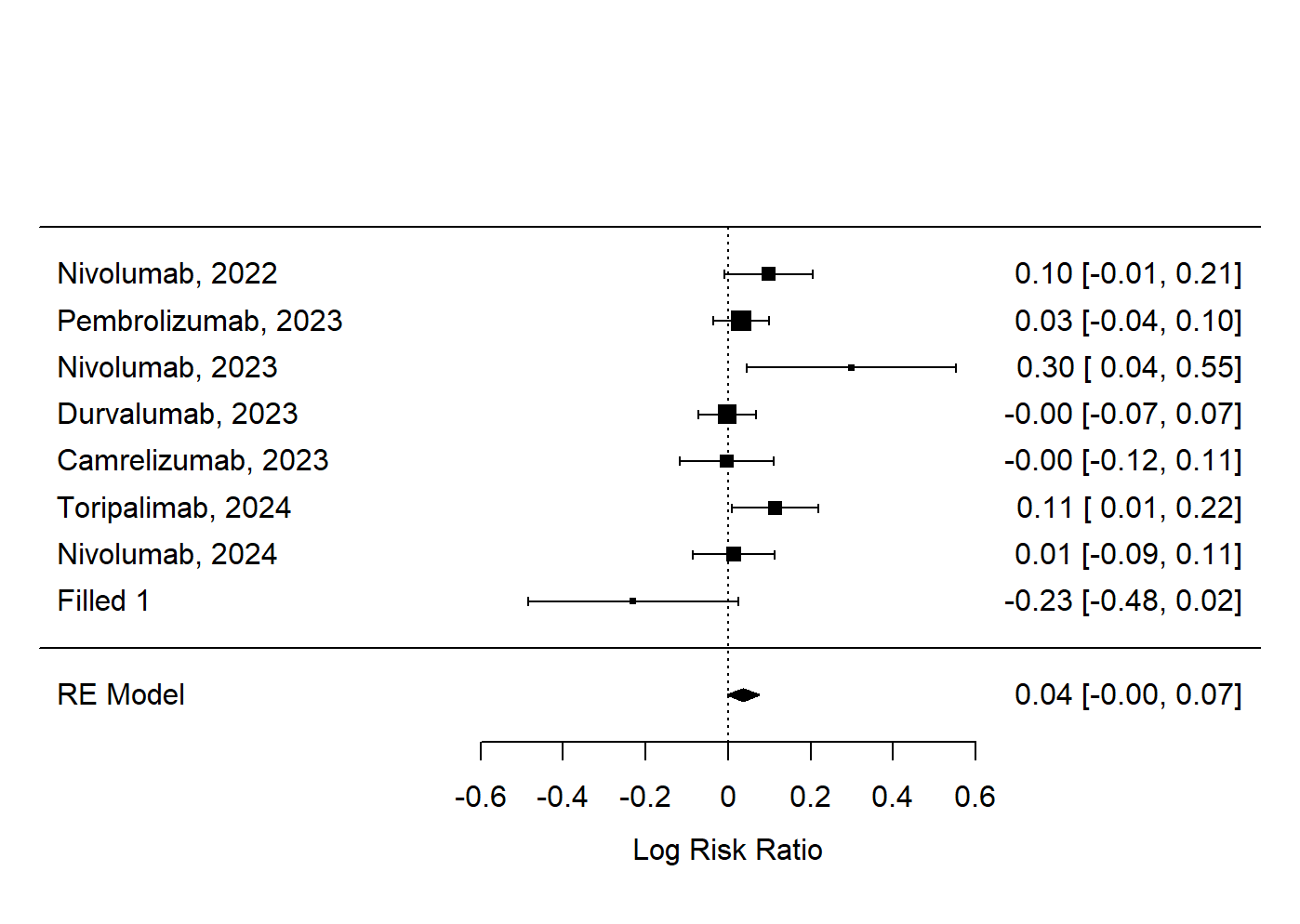


**Figure S13. The outcome of the Trimfill method of Surgery rate.**


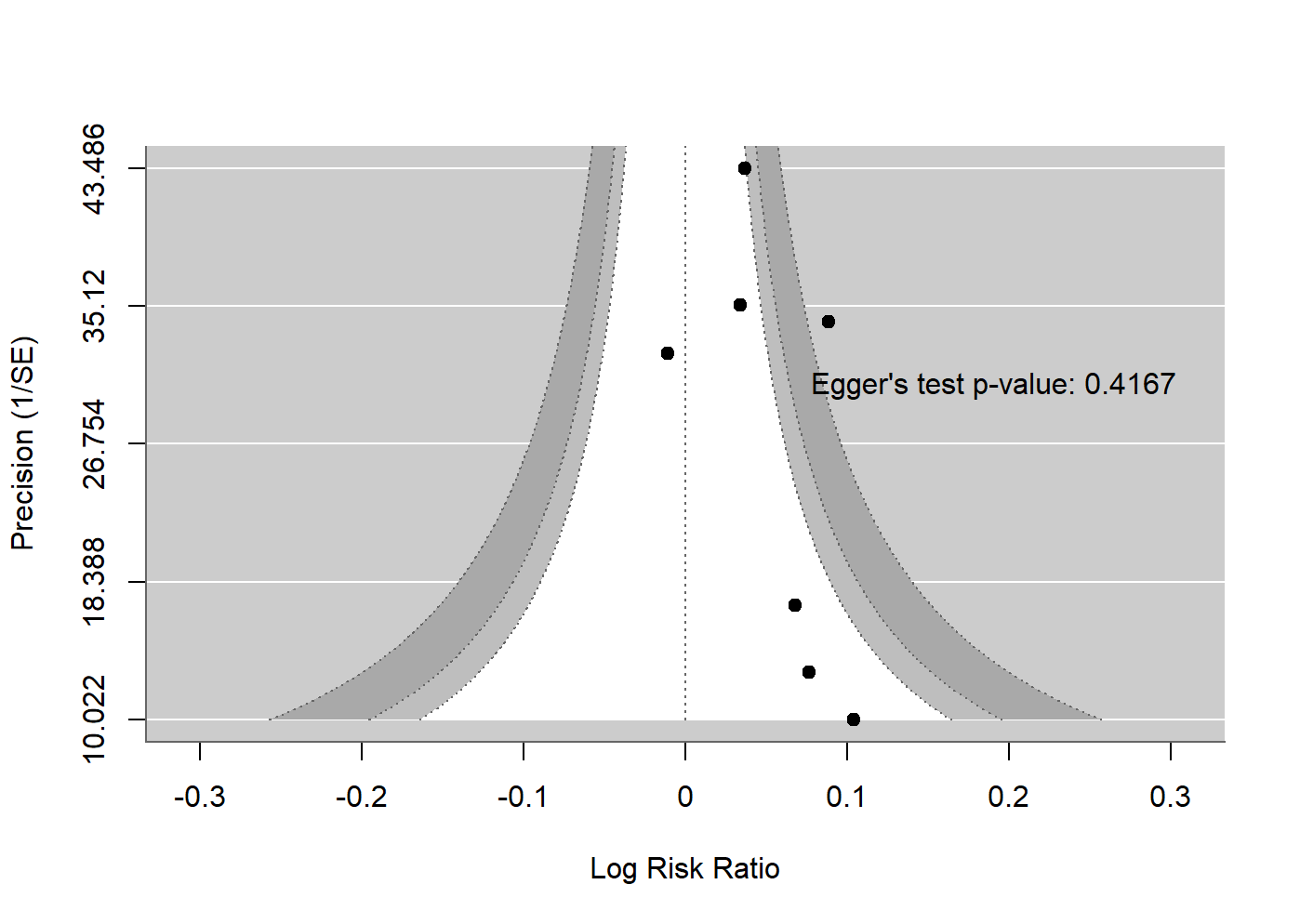


**Figure S14. Funnel plot and Egger’s regression of R0 resection.**


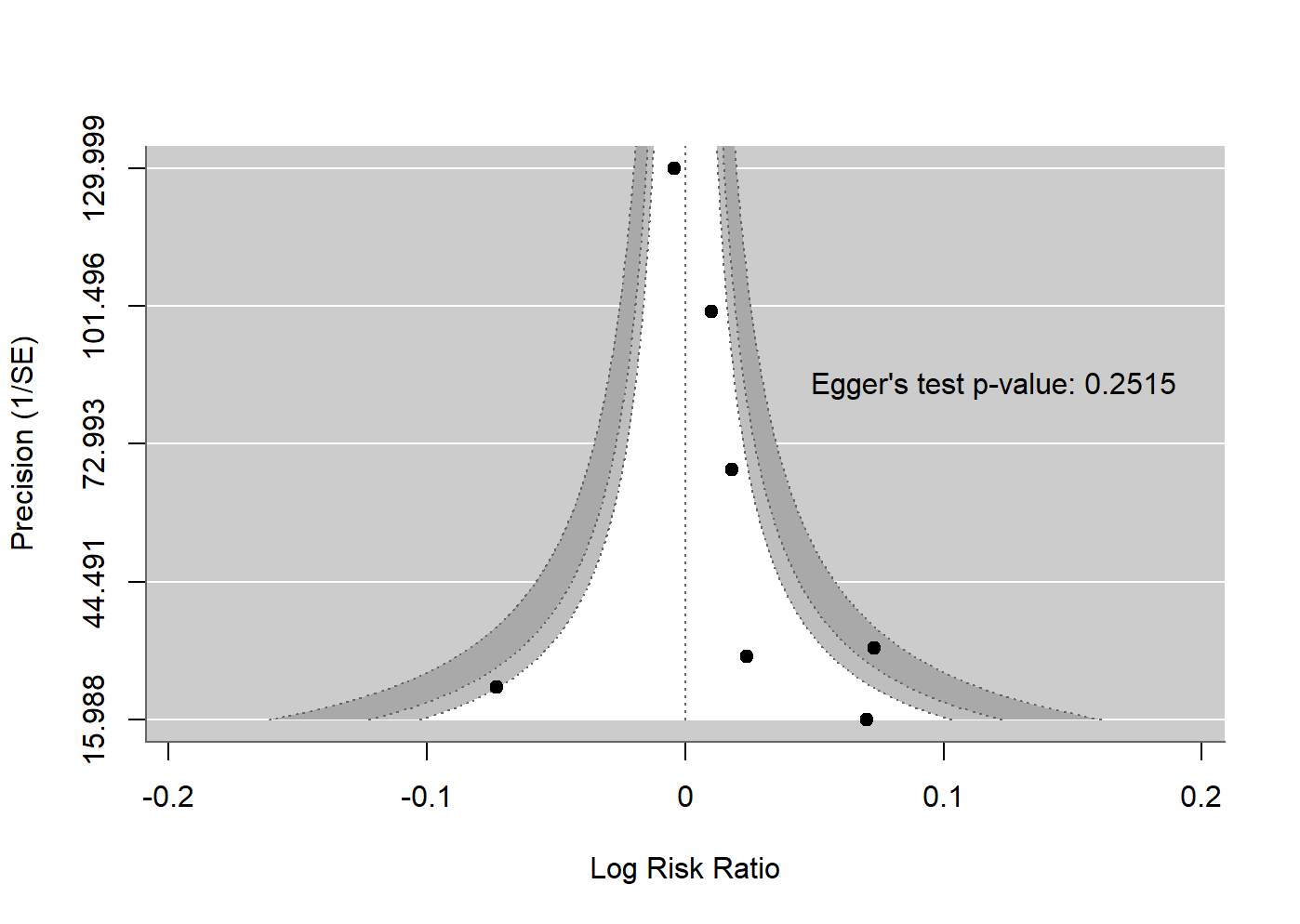


**Figure S14. Funnel plot and Egger’s regression of All TRAEs.**

**
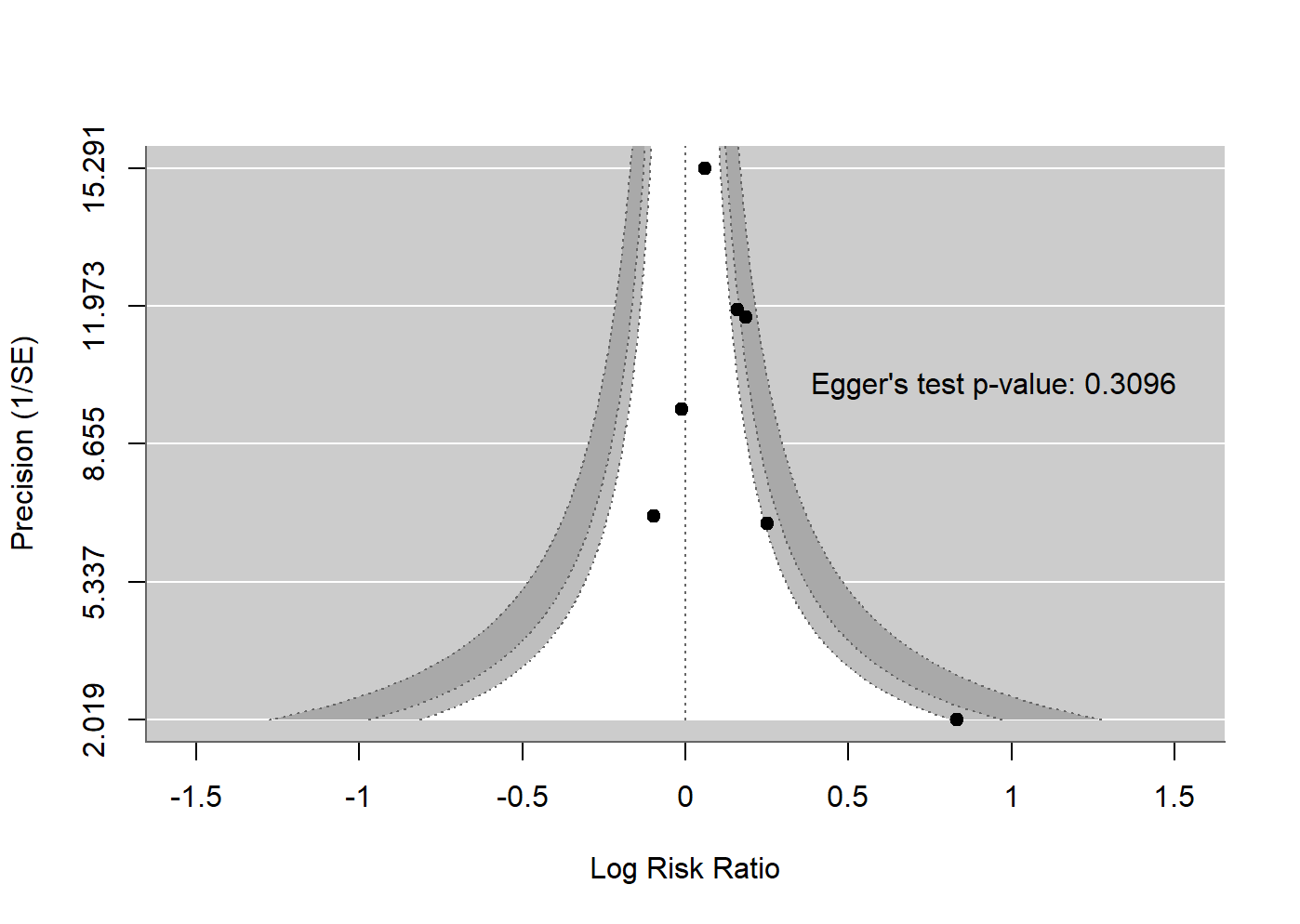
**

**Figure S15. Funnel plot and Egger’s regression of TRAE>Grade 3.**

**
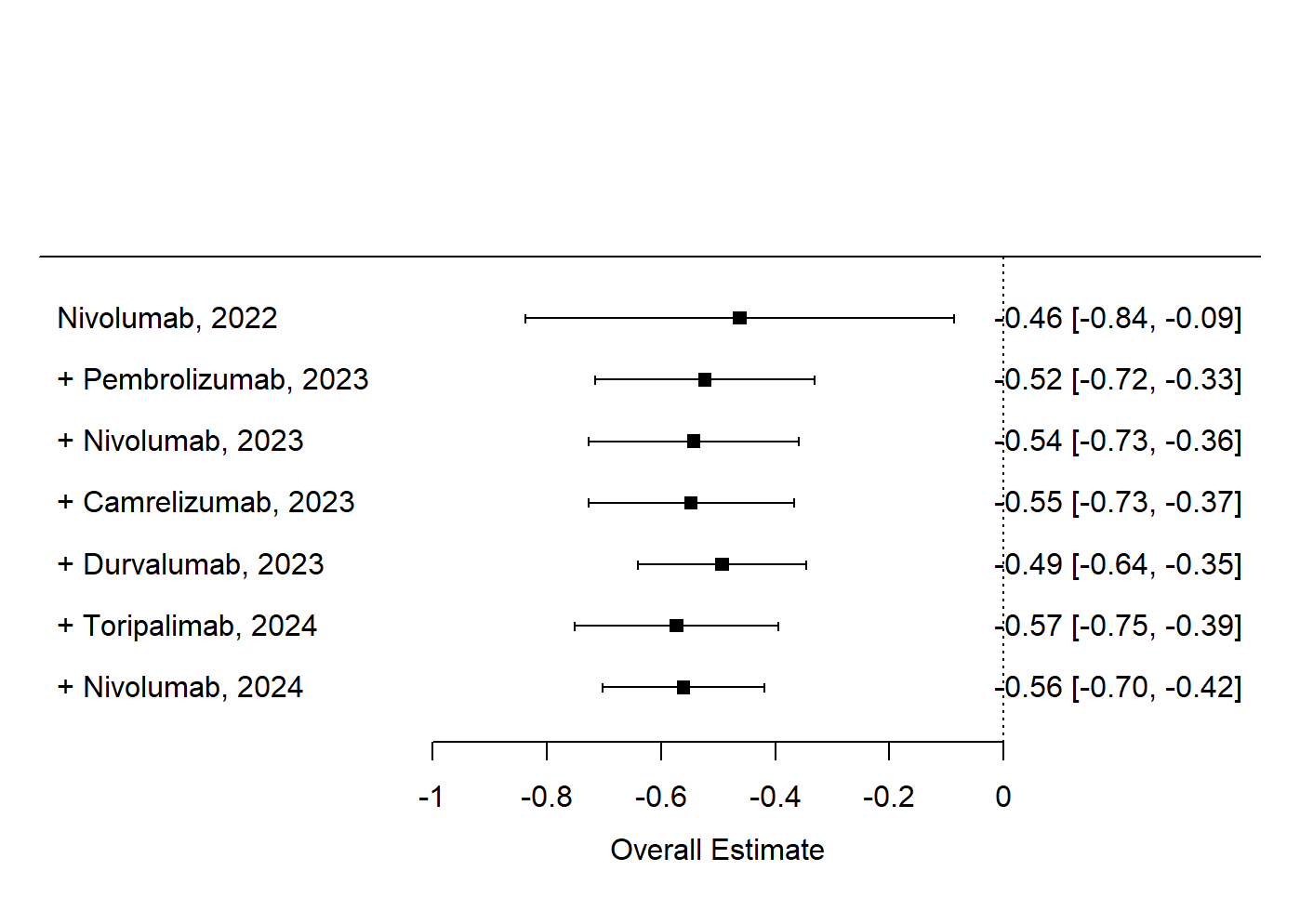
**

**Figure S16. Results of sensitivity analysis by the cumulative method of EFS.**

**
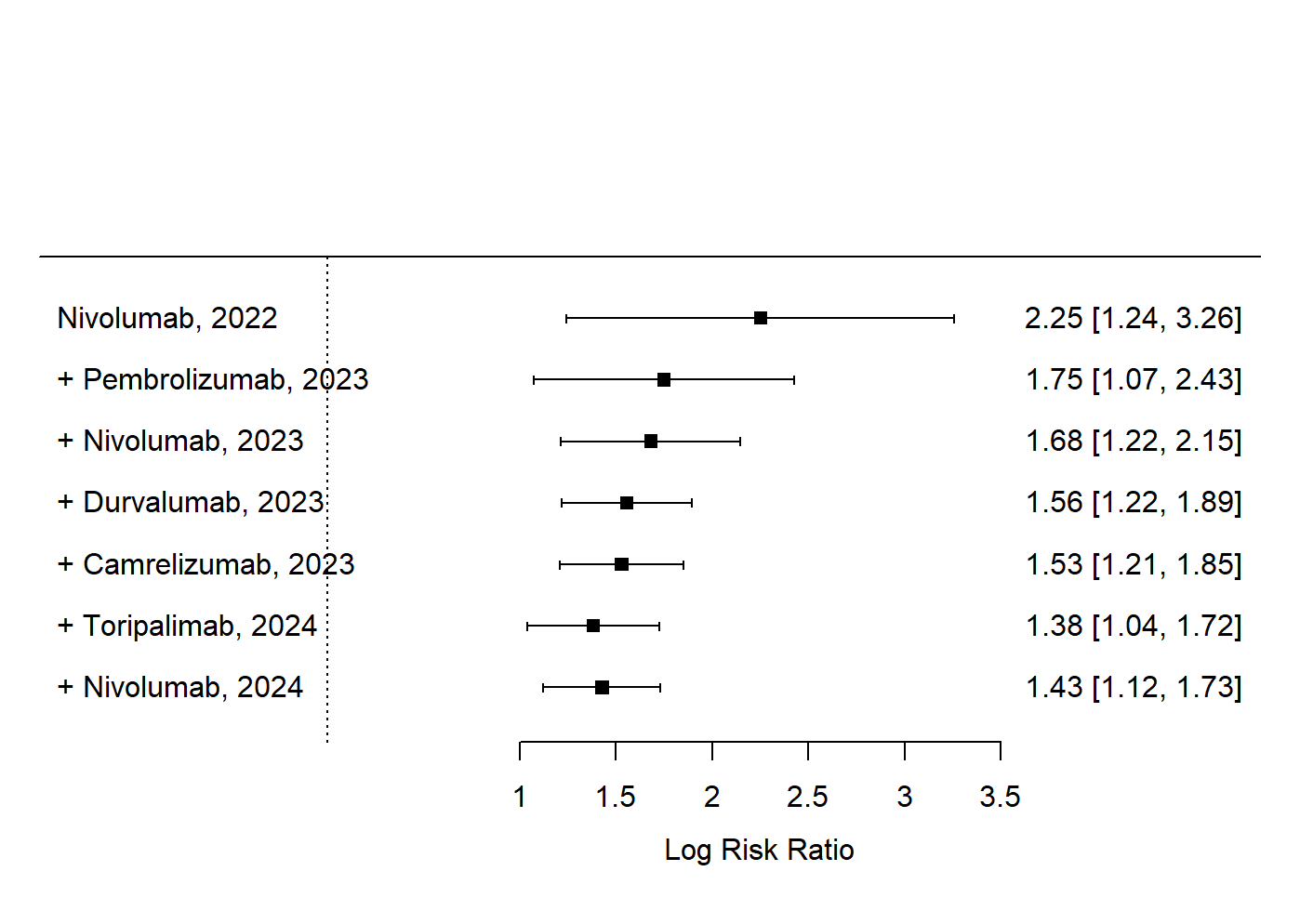
**

**Figure S17. Results of sensitivity analysis by the cumulative method of pCR.**


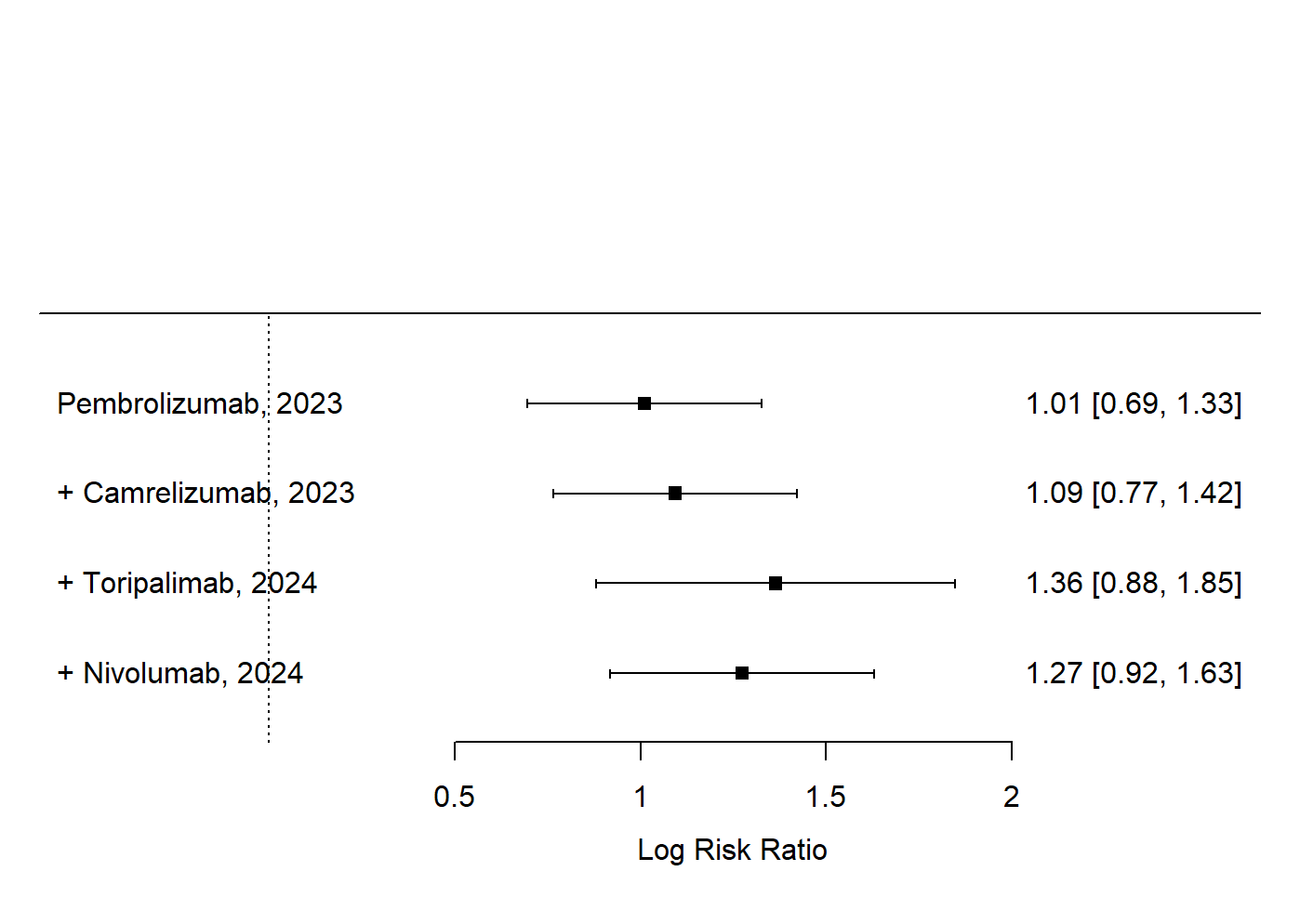


**Figure S18. Results of sensitivity analysis by the cumulative method of MPR.**


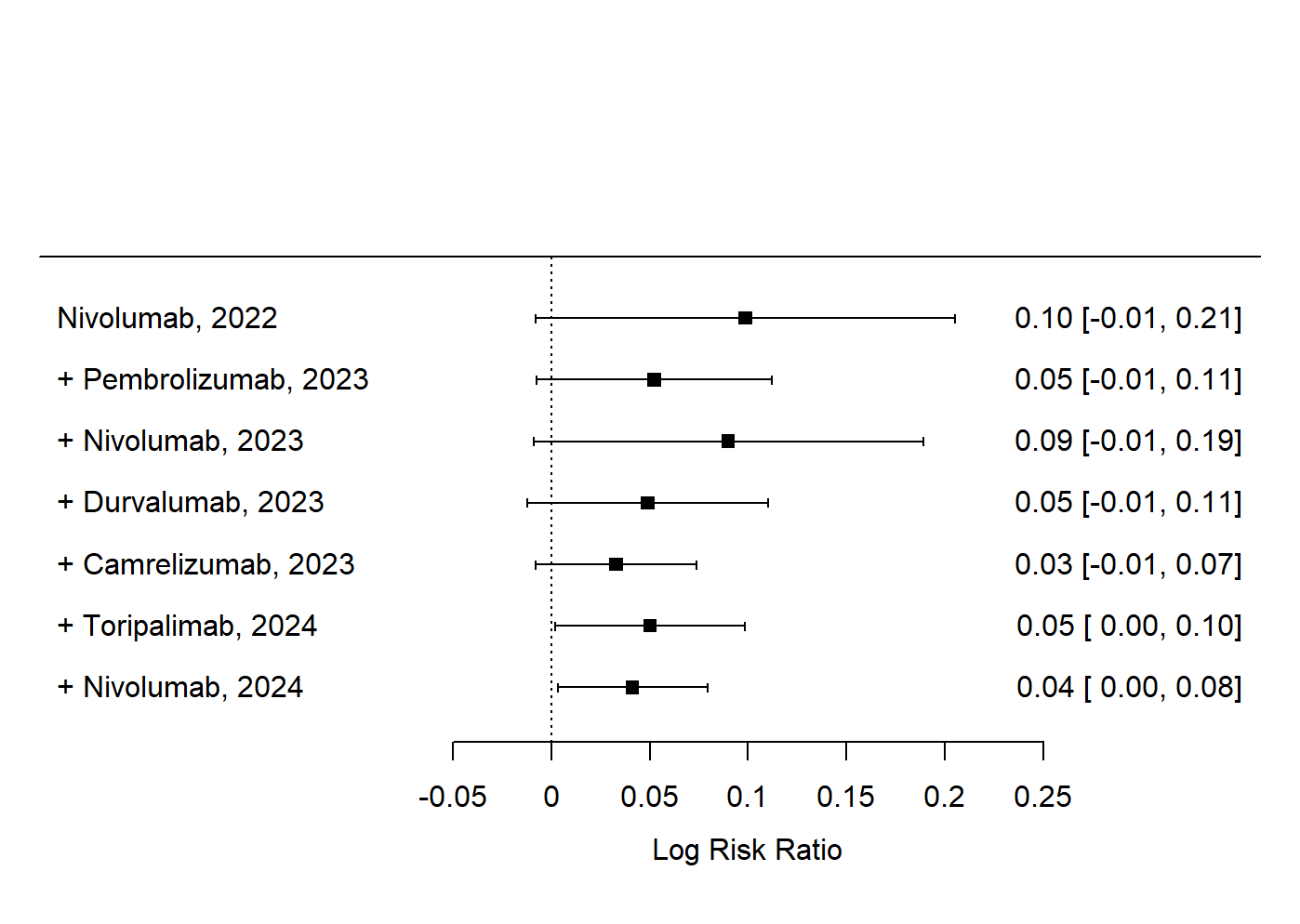


**Figure S19. Results of sensitivity analysis by the cumulative method of Surgery rate.**


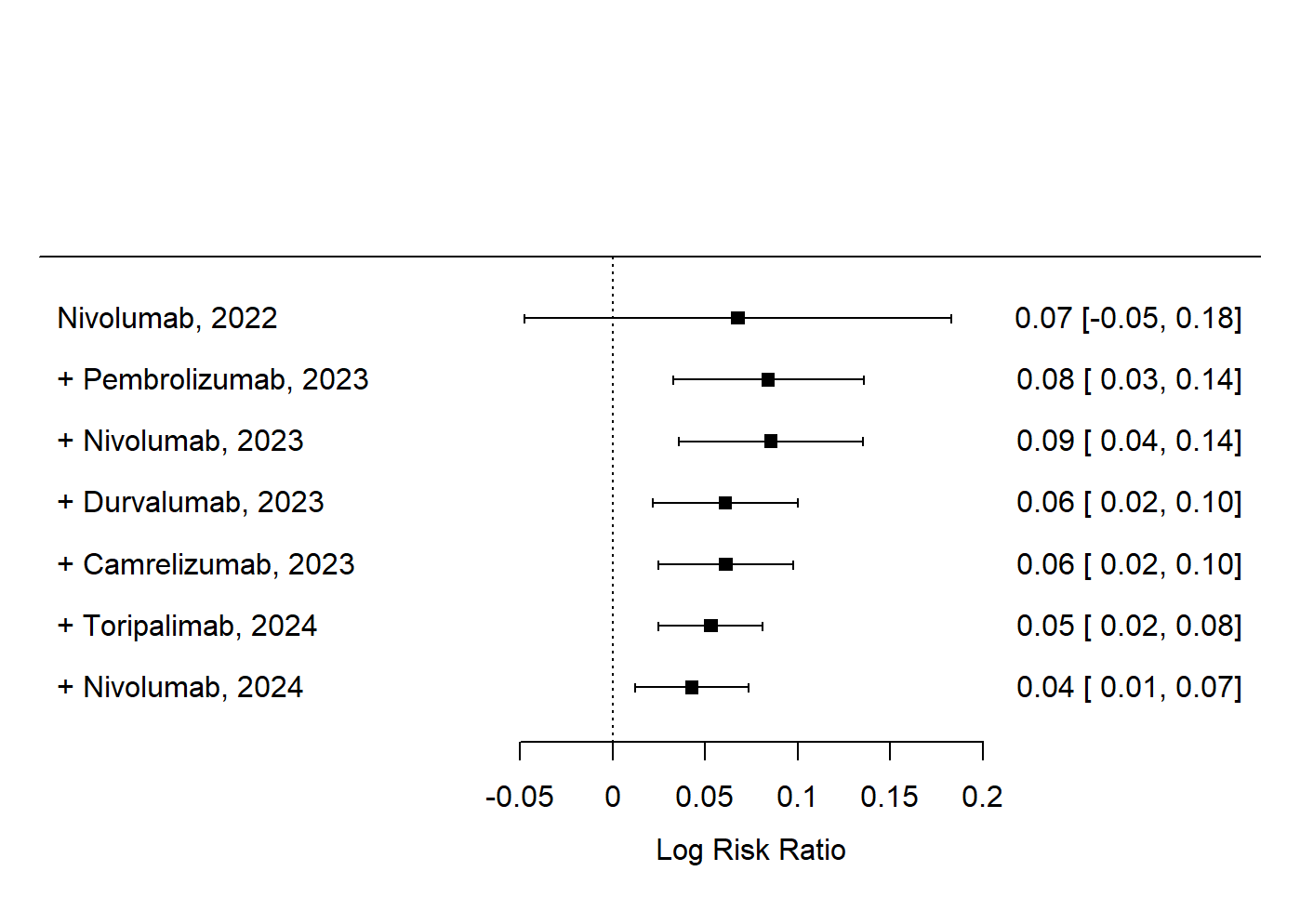


**Figure S20. Results of sensitivity analysis by the cumulative method of R0 resection.**


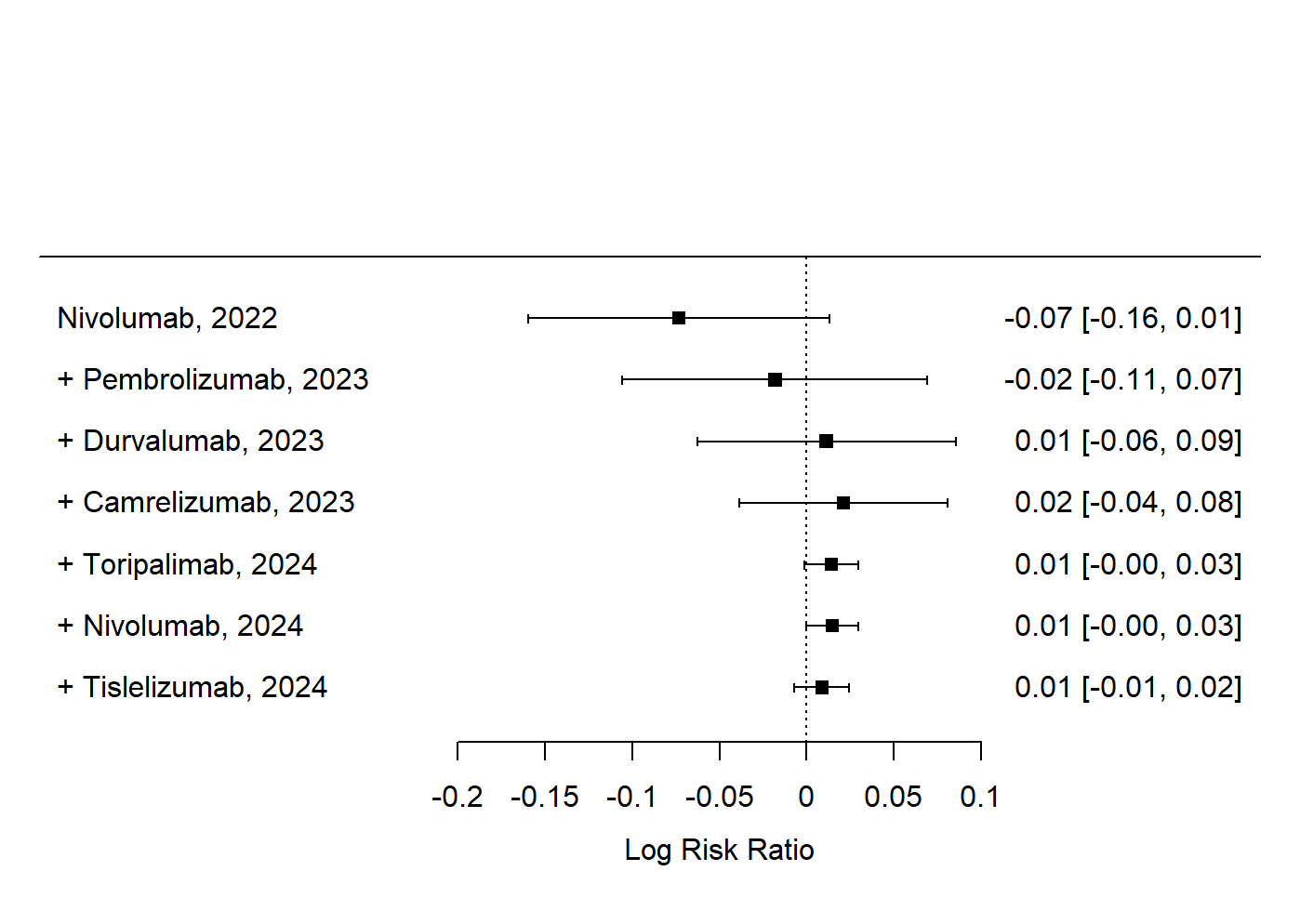


**Figure S21. Results of sensitivity analysis by the cumulative method of All TRAES.**


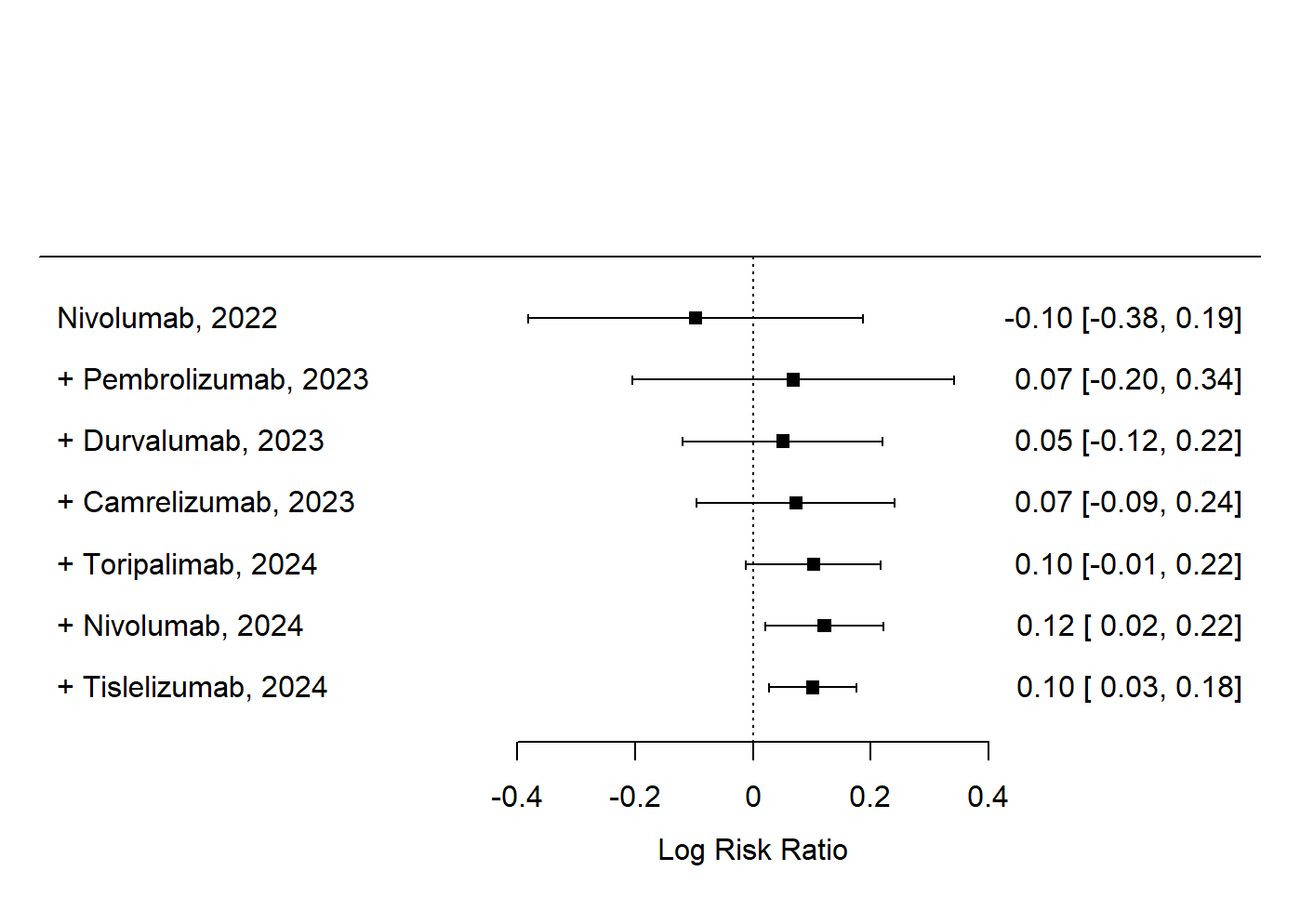


**Figure S22. Results of sensitivity analysis by the cumulative method of TRAE >Grade 3.**

**Table S3. Risk of Bias**

**
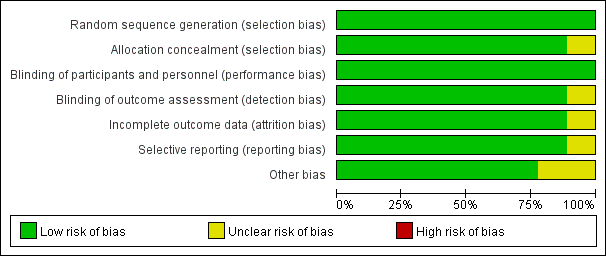

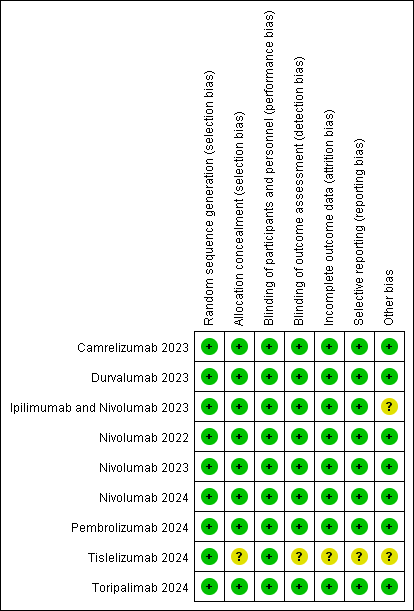
**

**Table S4. Results of the GRADE Classification.**
